# A Central Role for Carbon-Overflow Pathways in the Modulation of Bacterial Cell Death

**DOI:** 10.1371/journal.ppat.1004205

**Published:** 2014-06-19

**Authors:** Vinai Chittezham Thomas, Marat R. Sadykov, Sujata S. Chaudhari, Joselyn Jones, Jennifer L. Endres, Todd J. Widhelm, Jong-Sam Ahn, Randeep S. Jawa, Matthew C. Zimmerman, Kenneth W. Bayles

**Affiliations:** 1 Center for Staphylococcal Research, Department of Pathology and Microbiology, University of Nebraska Medical Center, Omaha, Nebraska, United States of America; 2 Cellular and Integrative Physiology, University of Nebraska Medical Center, Omaha, Nebraska, United States of America; 3 Department of Surgery, Stony Brook University School of Medicine, Stony Brook, New York, United States of America; National Institute of Allergy and Infectious Diseases, National Institutes of Health, United States of America

## Abstract

Similar to developmental programs in eukaryotes, the death of a subpopulation of cells is thought to benefit bacterial biofilm development. However mechanisms that mediate a tight control over cell death are not clearly understood at the population level. Here we reveal that CidR dependent pyruvate oxidase (CidC) and α-acetolactate synthase/decarboxylase (AlsSD) overflow metabolic pathways, which are active during staphylococcal biofilm development, modulate cell death to achieve optimal biofilm biomass. Whereas acetate derived from CidC activity potentiates cell death in cells by a mechanism dependent on intracellular acidification and respiratory inhibition, AlsSD activity effectively counters CidC action by diverting carbon flux towards neutral rather than acidic byproducts and consuming intracellular protons in the process. Furthermore, the physiological features that accompany metabolic activation of cell death bears remarkable similarities to hallmarks of eukaryotic programmed cell death, including the generation of reactive oxygen species and DNA damage. Finally, we demonstrate that the metabolic modulation of cell death not only affects biofilm development but also biofilm-dependent disease outcomes. Given the ubiquity of such carbon overflow pathways in diverse bacterial species, we propose that the metabolic control of cell death may be a fundamental feature of prokaryotic development.

## Author Summary

Many bacterial species including the pathogen *Staphylococcus aureus* are capable of adhering to surfaces and forming complex communities called biofilms. This mode of growth can be particularly challenging from an infection control standpoint, as they are often refractory to antibiotics and host immune system. Although developmental processes underlying biofilm formation are not entirely clear, recent evidence suggests that cell death of a subpopulation is crucial for its maturation. In this study we provide insight regarding the metabolic pathways that control cell death and demonstrate that acetate, a by-product of glucose catabolism, potentiates a form of cell death that exhibits physiological and biochemical hallmarks of apoptosis in eukaryotic organisms. Finally, we demonstrate that altering the ability of metabolic pathways that regulate acetate mediated cell death in *S. aureus* affects the outcome of biofilm-related diseases, such as infective endocarditis.

## Introduction

The balanced progression of cell division and apoptotic events is a classic hallmark of eukaryotic development [1]. Intriguingly, a similar homeostatic control of cell death, lysis and proliferation is predicted to benefit the development of adherent multicellular bacterial assemblages (called biofilms) by providing nutrients and critical biofilm building matrix components like extracellular DNA (eDNA) [2], [3]. Consistent with this assumption, recent investigations have revealed that bacteria, like eukaryotes not only harbor elaborate regulatory systems that modulate cell death, but also display biochemical and physiological hallmarks characteristic of programmed cell death (PCD) [4], [5], [6].

The molecular components that mediate cell death in *S. aureus* are regulated, in part, by the LysR-type transcriptional regulator, CidR [7] and include a set of membrane bound proteins, CidA and CidB, whose functions are predicted to be analogous to the Bcl-2 family of apoptotic modulators in eukaryotes [2], [3]. However, less clear are the mechanistic contributions of other members of the CidR regulon in cell death, specifically those enzymes that are active during overflow metabolism, pyruvate oxidase (CidC) and α-acetolactate synthase/decarboxylase (AlsSD) [8], [9]. Given that these enzymes are the only additional members of the CidR regulon and that multiple physiological signals that directly affect both central metabolism and cell senescence coordinate their expression [10], we predicted an intricate role for these proteins in the physiology of cell death.

Here, we report that both CidC and AlsSD carbon-overflow pathways contribute to staphylococcal cell death. Our results demonstrate that cell death is potentiated by acetate, a major weak acid byproduct of glucose catabolism, whose levels are antithetically modulated by CidC and AlsSD activities. We also report that the physiological features accompanying staphylococcal cell death resemble eukaryotic PCD (apoptosis) wherein cell death is associated with respiratory dysfunction, increased ROS production and DNA damage. Finally, we demonstrate a role for staphylococcal PCD in biofilm development and pathogenesis.

## Results

### Glucose-dependent cell death in stationary phase exhibits hallmarks of PCD

**Figure 1:**
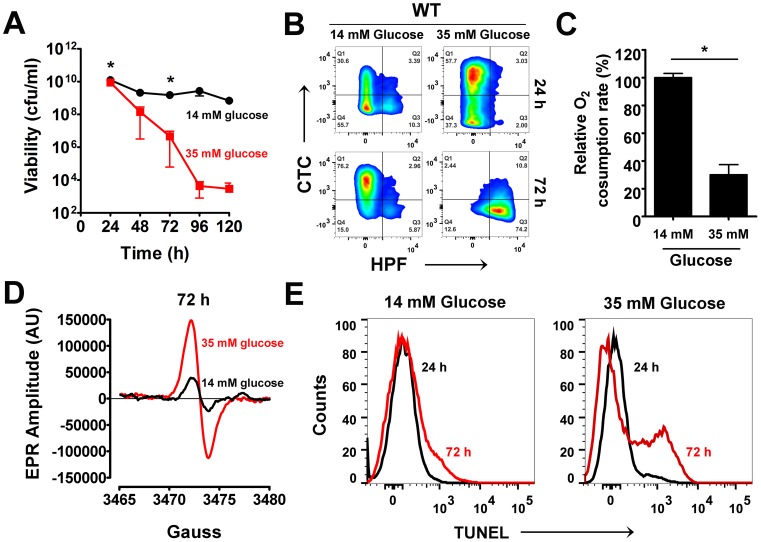
Excess glucose promotes cell death in *S. aureus*. (**A**) *S. aureus* UAMS-1 cell viabilities (cfu/ml, mean ± SD) were monitored every 24 h over a period of five days in TSB-14 mM or TSB-35 mM glucose. (*) indicates time-points when samples were withdrawn for flow cytometry and electron paramagnetic resonance spectroscopy (EPR) analysis. (**B**) Psuedocolor density plots of cells double stained with HPF and CTC. (**C**) Oxygen consumption rates were determined using a commercially available kit (* *P*<0.05, Students *t*-test, n = 2) (**D**) Whole cell EPR analysis of *S. aureus* undergoing programmed cell death (PCD) was carried out after 72 h growth. (**E**) TUNEL staining of 1% paraformaldehyde fixed cells was performed using the ApoDirect kit (Millipore) and analyzed by flow cytometry. Gates were based on 24 h cultures that exhibited minimal loss in cell viability.

Multiple studies have linked the uptake and metabolic fate of glucose to the regulation of PCD during eukaryotic development [11]. To determine whether such correlations are broadly conserved in bacteria, the effects of glucose on staphylococcal cell death were assessed over a period of five days by monitoring the colony forming units (cfu/ml) of wild-type cells grown aerobically in rich media (tryptic soy broth, TSB) containing either 14 mM or 35 mM glucose. Although there appeared to be no significant difference in the viable cell counts after 24 h of growth in either type of media, subsequent stationary phase survival of wild-type cells was dependent on initial glucose concentrations wherein *S. aureus* grown in TSB-35 mM glucose displayed a steep decline in viability (∼7 log_10_ difference) compared to the modest decline (∼1.2 log_10_) observed for cells grown in TSB-14 mM glucose over the same period of time (120 h) (Fig. 1A). These results indicate that growth in excess glucose reduced the survival of *S. aureus* in stationary phase.

To ascertain whether excess glucose-mediated staphylococcal cell death bore similarities to PCD, we explored the physiological status of the dying population by flow cytometry after 72 h of growth and compared them to a 24 h reference point when cells were relatively healthy based on viable counts. Respiratory potential was estimated using the cell permeable redox dye, cyano-2,3-ditolyl tetrazolium chloride (CTC). Reduction of CTC into a red insoluble fluorescent formazan that accumulates intracellularly is achieved by dehydrogenases of the electron transport chain (ETC) that are expressed within actively respiring bacterial populations [12]. In addition to CTC, cells were co-stained with 3′-(*p*-hydroxyphenyl) fluoroscein (HPF), a cell permeable fluorescent reporter that has widely been used to detect levels of highly reactive oxygen species like hydroxyl radicals (OH^•^) [13]. CTC staining of *S. aureus* grown in TSB-14 mM glucose revealed a healthy respiring sub-population (∼34%) at 24 h and a relatively smaller population of cells (13%) undergoing oxidative stress (Fig. 1B, Table S1). By 72 h the respiring population under these very same conditions increased to 79% (Fig. 1B, Table S1). This is expected as dependency on the TCA cycle and oxidative phosphorylation for cellular energetic needs increases in stationary phase, upon exhaustion of glucose from the media. Interestingly, *S. aureus* grown in TSB-35 mM glucose revealed an even larger population (∼61%) that reduced CTC at 24 h when compared to 14 mM glucose (Fig. 1B, Table S1). The increased reduction of CTC under these conditions could not have resulted from a corresponding increase in the rate of cellular respiration, as the ability of these cells to consume oxygen as a terminal electron acceptor had significantly decreased (Fig. 1C). These observations suggest that aerobic growth in excess glucose not only results in the inhibition of respiration, but may also promote the promiscuous transfer of electrons to alternate acceptors like CTC, due to a bottleneck in the ETC.

The transfer of electrons via a functional ETC has previously been proposed to ameliorate oxidative stress by curtailing the single electron reduction of oxygen to superoxide radicals (O_2_^•−^), a precursor of the highly reactive hydroxyl radical (OH^•^) [14]. Hence, we argued that the decreased functionality of ETC observed for cells grown in excess glucose may eventually promote the production of ROS. Although we did not observe OH^•^ at 24 h of growth, we detected a dramatic increase of HPF stained cells by 72 h of growth in TSB-35 mM glucose but not in TSB-14 mM glucose (Fig. 1B, Table S1). The temporal production of ROS was confirmed by electron paramagnetic resonance (EPR) spectroscopic analysis of samples incubated with the spin probe, 1-hydroxy-methoxycarnonyl-2,2,5,5-tetramethyl-pyrrolidine hydrochloride (CM-H). Cells grown in TSB-35 mM glucose exhibited approximately 3-fold increase in EPR peak amplitude by 72 h relative to those grown in TSB-14 mM glucose (Fig. 1D). To determine the chemical nature and relative levels of various ROS produced, we incubated samples with either superoxide dismutase (SOD; O_2_^•−^ scavenger) or dimethyl thiourea (DMTU; OH^•^ scavenger) prior to the addition of CM-H. This approach revealed that cells undergoing cell death produced both superoxide and hydroxyl radicals (Fig. S1).

An abundance of cellular ROS mediates several types of DNA damage, including single and double stranded breaks that lead to DNA fragmentation [15]. We performed TUNEL (terminal deoxynucleotidyl transferase dUTP nick end labeling) assays on *S. aureus* undergoing oxidative stress to estimate the population of cells undergoing DNA fragmentation by flow cytometry. Consistent with the temporal pattern of ROS production observed earlier, we detected a sub-population of cells with fragmented DNA (TUNEL positive) by 72 h of growth under excess glucose conditions (Fig. 1E). Notably, only minimal TUNEL staining (Fig. 1E) was detected after 72 h for cultures supplemented with 14 mM glucose. Collectively, these observations suggest that cell death resulting from growth under excess glucose exhibits multiple hallmarks of eukaryotic PCD. Interestingly, the phenotypic hallmarks of PCD were not restricted to growth of *S. aureus* under glucose rich conditions alone, but were also observed when grown in the presence of excess fructose, mannitol and sucrose suggesting a strong association between carbon catabolism and cell death (Fig. S2).

### Acetate potentiates cell death under low pH

**Figure 2:**
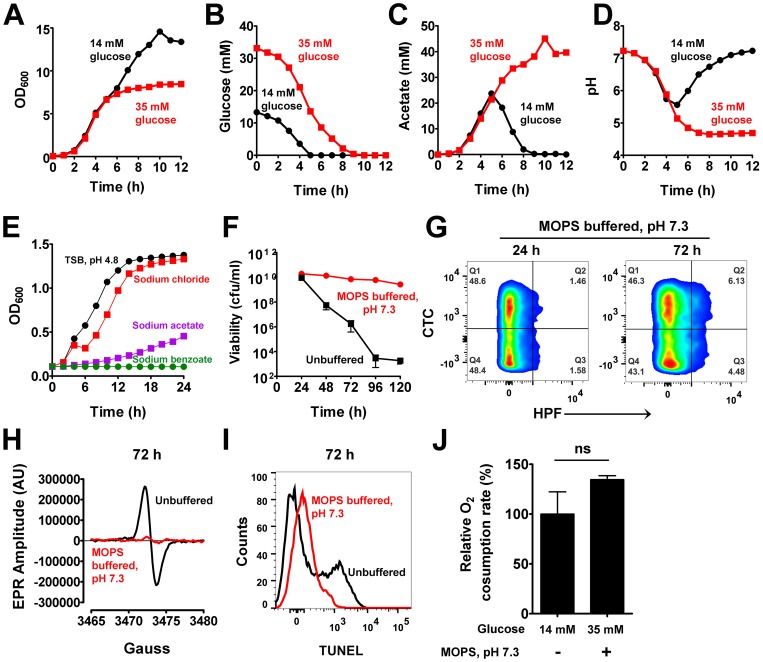
Acetate potentiates cell death. (**A**) Aliquots of *S. aureus* UAMS-1 were sampled over time and their O.D._600_ determined. Depletion of glucose (**B**), acetate levels (**C**) and pH of culture supernatants (**D**) were determined at the indicated times. (**E**) The growth inhibitory effects of acetate under low pH were assessed in TSB buffered at pH 4.8 with 30 mM HOMOPIES. Various salts including sodium chloride, sodium acetate, and sodium benzoate were supplemented to the media at a final concentration of 50 mM and growth monitored (n = 3, mean ± SD). Stationary phase viability (**F**), CTC/HPF double staining (**G**), EPR spectroscopy analysis (**H**), TUNEL staining (**I**) and respiratory capacity (**J**) of 72-hour cultures were determined following growth in TSB-35 mM glucose buffered with 50 mM MOPS, pH 7.3.

How does excess glucose mediate cell death? It is well known that *S. aureus* cultured in excess glucose undergoes CcpA-mediated catabolite repression [16]. This ensures that acetate accumulates in the media as a byproduct of glucose catabolism. However, once glucose is completely exhausted from the media, the TCA cycle is progressively relieved of CcpA repression and excreted acetate is oxidized to generate energy required for subsequent growth [16]. Growth of *S. aureus* in TSB-14 mM glucose displayed such a classic diauxie, where glucose was consumed within 5 h and subsequent growth was dependent on the consumption of acetate by 9 h (Fig. 2A–C). The temporal dynamics of acetate levels in the media were also reflected in the pH shift of the culture supernatant from 7.2 to 5.5 during acetate accumulation and from 5.5 to 7.2 during its depletion (Fig. 2C, 2D). As observed previously [17], growth of *S. aureus* in TSB-35 mM glucose did not display the expected diauxic shift (Fig. 2A). Although excess glucose was consumed within 9 h, acetate remained unutilized and the pH of the culture was maintained at 4.6 (Fig. 2B–D).

Based on these observations, we hypothesized that cells grown in excess glucose would eventually be inhibited by high concentrations of acetate and low pH. The growth inhibitory effects of weak organic acids like acetate are largely dependent on pH [18]. As the extracellular pH nears the pK*a* of acetate (pK*a* = 4.76), the protonated (uncharged) membrane permeant form of the acid (CH_3_COOH) replaces its corresponding ionic forms (CH_3_COO^−^; H^+^), thus allowing the former species to passively breach bacterial membranes and dissociate within their relatively neutral cytoplasm [18]. If left unchecked, such an event may lead to growth inhibition and lethality through cytoplasmic acidification [18]. Given that the pK*a* of acetate is easily met during growth in excess glucose, it seemed plausible that this acidic metabolite may represent a physiological trigger for cell death. To test whether acetate was capable of inhibiting *S. aureus* growth under acidic conditions, we buffered TSB at pH 4.8 using 30 mM HOMOPIPES (homopiperazine-N,N′-bis-2-(ethanesulfonic acid) and challenged cultures with the sodium salt of acetate. As expected, acetate inhibited the growth of *S. aureus* under these conditions, but neither acidic pH alone nor addition of an equimolar concentration of sodium chloride (50 mM) inhibited growth to the same extent as sodium acetate (Fig. 2E). Further, the addition of a non-metabolizable weak acid, benzoate (pK*a* = 4.2) was as toxic as acetate under low pH (Fig. 2E). These observations suggest that acetate mediated growth inhibition is a direct consequence of intracellular acidification and not due to secondary metabolites resulting from intracellular catabolism of acetate.

To confirm that cell death is dependent on the weak acid properties of acetate, we grew *S. aureus* in TSB-35 mM glucose that was buffered to a pH of 7.3 with 50 mM MOPS (3-(N-morpholino) propanesulfonic acid). We reasoned that although cells would utilize excess glucose to generate acetate, the relatively neutral pH of the medium would allow it to remain in the ionic state and prevent it from permeating and acidifying the interior of cells. Indeed as predicted, *S. aureus* under these conditions did not undergo cell death despite its growth in excess glucose (Fig. 2F). Remarkably, we also observed a dramatic reduction in the generation of ROS (Fig. 2G–H, Table S1), decreased DNA damage (Fig. 2I) and comparable rates of respiration relative to wild-type (Fig. 2J) under these conditions, suggesting that excess glucose per se was not responsible for the phenotypes associated with cell death. Rather these observations collectively demonstrate that acetate, a metabolic byproduct of glucose catabolism triggered these phenotypes under acidic pH.

### CidR-dependent overflow metabolic pathways regulate staphylococcal cell death

**Figure 3:**
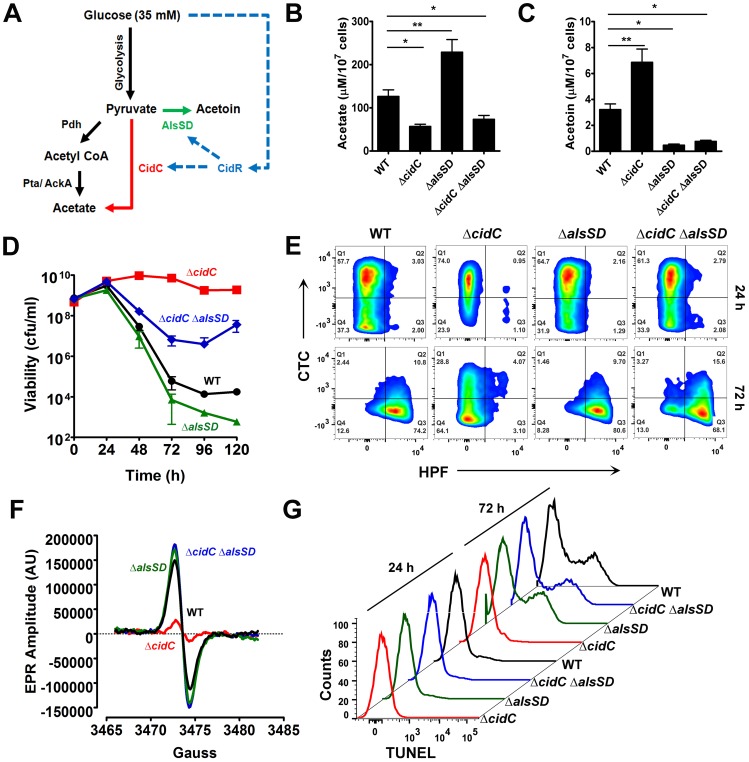
CidC and AlsSD carbon overflow pathways modulate cell death. (**A**) Schematic of overflow metabolic pathways controlled by the LysR-type transcriptional regulator, CidR. Under aerobic conditions and in the presence of glucose, acetyl CoA derived from the decarboxylation of pyruvate by Pdh (pyruvate dehydrogenase) is converted to acetate by the Pta (phosphotransacetylase)/AckA (acetate kinase) pathway (black). Excess glucose can activate additional metabolic pathways (CidR regulated; blue) that divert pyruvate towards the production of acetate (red) and the neutral metabolite, acetoin (green) through CidC (pyruvate oxidase) and AlsSD (α-acetolactate synthase/α-acetolactate decarboxylase) enzyme activities, respectively. The concentrations of acetate (**B**) and acetoin (**C**) were determined from culture supernatants of various *S. aureus* strains after 24 h of growth in TSB-35 mM glucose using commercially available kits (R-Biopharm, Germany). Statistical significance was assessed using one way ANOVA followed by Newman Kewl's multiple post-comparison test, n = 3; * *P*<0.05, ** *P*<0.005). Stationary phase cell viability (**D**), flow cytometry of HPF/CTC double stained samples (**E**), EPR analysis (**F**) and TUNEL staining (**G**) were determined following aerobic growth of cultures in TSB-35 mM glucose.

Although acetate is primarily produced in *S. aureus* by the phosphotransacetylase (Pta)-acetate kinase (AckA) pathway, this metabolic pathway is unlikely to be directly involved in cell death as its activity is evident even during growth in 14 mM glucose, a condition where cell death is not triggered [19]. Additionally, disruption of this pathway surprisingly enhanced the rate of cell death during growth despite a decrease in acetate production [19]. This led us to reason that acetate-dependent cell death must be controlled by an alternate pathway, such as CidC. The *cidC* gene encodes a pyruvate oxidase that directly converts pyruvate to acetate and carbon dioxide and its expression is partly under the control of the regulator, CidR, whose activity is up-regulated in response to excess glucose [7], [19]. As the *alsSD* metabolic operon that results in the conversion of pyruvate to acetoin is also co-regulated by CidR, we hypothesized that both these pathways may modulate acetate-dependent cell death by competition for their common substrate, pyruvate, under conditions of excess glucose (Fig. 3A).

To test this hypothesis, we initially determined the levels of acetate and acetoin in 24 h culture supernatants of both Δ*cidC* and Δ*alsSD* mutants relative to WT. Compared to the wild-type strain, the Δ*cidC* mutant grown in TSB-35 mM glucose accumulated less acetate and relatively higher levels of acetoin (Fig. 3B, 3C). Conversely the Δ*alsSD* mutant excreted an excess of acetate (Fig. 3B) suggesting that both pathways competitively displaced pyruvate. We then tested the effects of *cidC* and *alsSD* pathways on cell death. In agreement with earlier studies [17], [20], mutation of either of these pathways resulted in opposing survival trends in stationary phase. Accordingly, a metabolic block in CidC activity (Δ*cidC*) enhanced stationary phase survival, while that of AlsSD (Δ*alsSD*) resulted in an increased rate of cell death compared to the wild-type strain (Fig. 3D). Consistent with the increased survival of the Δ*cidC* mutant and in contrast to WT and the Δ*alsSD* mutant, HPF-CTC double staining of 72 h cultures revealed a healthy population of respiring cells that exhibited low levels of ROS (Fig. 3E, Table S1), a phenotype that was also confirmed by EPR spectroscopy (Fig. 3F). In addition, flow cytometry detected fewer TUNEL-positive cells in the Δ*cidC* mutant, suggesting decreased DNA damage in these cells (Fig. 3G). Indeed, the cell death phenotypes associated with both Δ*cidC* and Δ*alsSD* mutants could be complemented *in trans* (Fig. S3) confirming their role in modulating cell death. Taken together, these data support the hypothesis that both CidC and AlsSD pathways modulate cell death by controlling flux through the pyruvate node.

Surprisingly, the Δ*alsSD* mutant generated ROS and exhibited DNA damage at levels similar to the wild-type strain (Fig. 3E–G, Table S1) despite an increase in loss of viability (Fig. 3D). This raised the possibility that in addition to affecting excreted acetate levels, there may be additional mechanisms by which the Δ*alsSD* mutant regulates cell death. To test this hypothesis we constructed a double Δ*cidC*Δ*alsSD* mutant. We reasoned that if regulation of extracellular acetate by substrate competition was the primary mechanism by which AlsSD modulated cell death, then the Δ*cidC*Δ*alsSD* double mutant would phenocopy the Δ*cidC* mutant. However, our results clearly demonstrate that the Δ*cidC*Δ*alsSD* double mutant exhibited increased loss of viability (Fig. 3D), increased generation of ROS (Fig. 3E–F, Table S1) and excessive levels of DNA damage (Fig. 3G) than the Δ*cidC* mutant despite excreting comparable levels of acetate (Fig. 3B). Such an effect may occur if mutation of *alsSD* caused cells to be more susceptible to lower concentrations of weak acids in the culture media. Indeed, growth of Δ*alsSD* mutants challenged with acetate, lactate or pyruvate was more easily inhibited than wild-type (Fig. S4).

**Figure 4:**
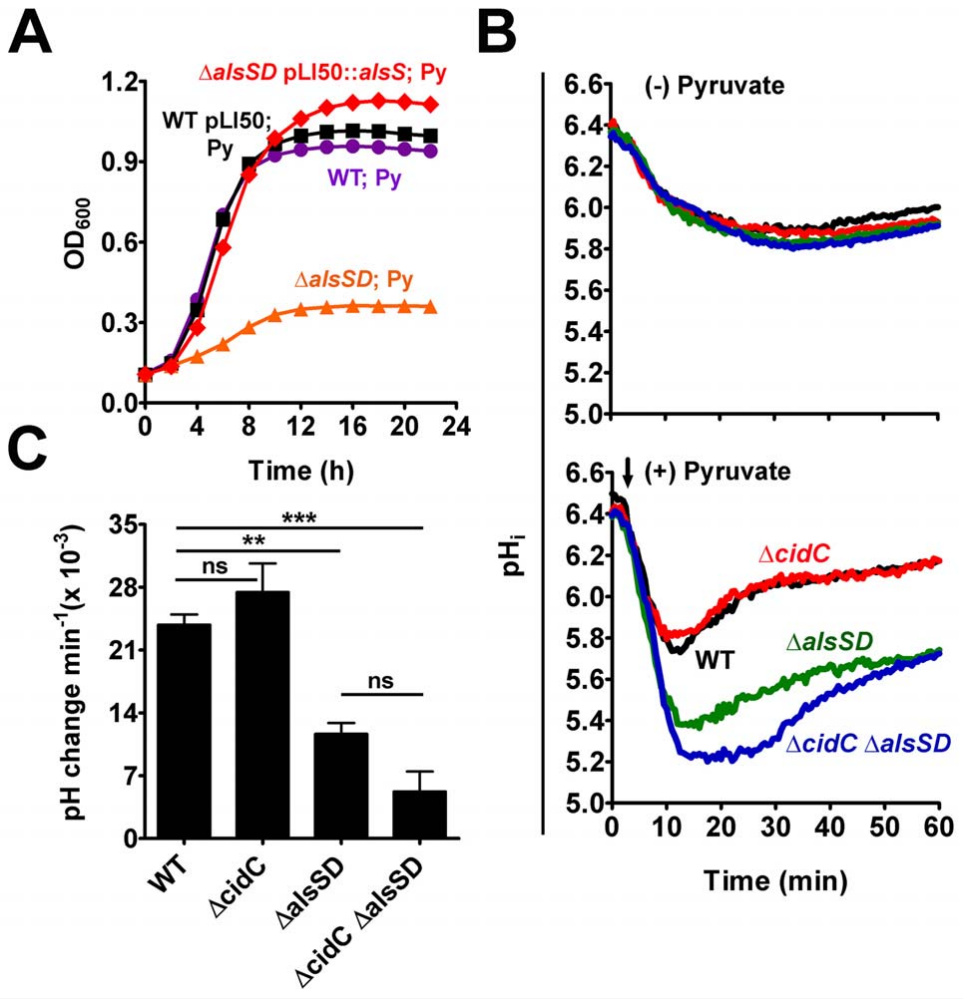
AlsSD activity counters cytoplasmic acidification. (**A**) Over-expression of AlsS rescues pyruvate-mediated growth inhibition of Δ*alsSD* mutant. (**B**) Intracellular pH (pHi) of UAMS-1 and derivatives following pyruvate stress. Bacterial cells loaded with the pH sensitive dye, cFSE, were suspended in potassium phosphate buffer (pH 4.5) and their pHi was measured as a function of the fluorescence emitted, either in the absence or presence of 40 mM sodium pyruvate (indicated by black arrow). (**C**) The rates of pHi recovery in each strain were measured from the slopes of the primary rise in pH_i_ traces represented in panel B, (Statistical significance was assessed using one way ANOVA followed by Newman Kewl's multiple post-comparison test, n = 3; ** *P*<0.005, *** *P*<0.0005).

We tested two plausible hypotheses to explain the observed hyper-susceptibility of Δ*alsSD* mutants to weak acid stress. First, we argued that the end product of AlsSD catabolism, acetoin, itself may be necessary to withstand weak acid stress as it is known to contribute to the maintenance of cellular redox status upon being converted to butanediol or serve as a carbon source during stationary phase of growth. To test this hypothesis we subjected the Δ*alsSD* mutant to pyruvic acid stress and asked whether supplementation of excess acetoin in culture could rescue the pyruvate-mediated growth inhibition of this mutant. Our results demonstrate that acetoin could not restore pyruvate-mediated growth inhibition of the Δ*alsSD* mutant (Fig. S5). Furthermore, transformation of the Δ*alsSD* mutant with a plasmid bearing the *alsS* gene alone (in the absence of its cognate partner, *alsD*) under the control of its native promoter was able to rescue this mutant from pyruvic acid-mediated stress to growth rates comparable to the parental control, thus excluding any role for acetoin in promoting weak acid resistance (Fig. 4A).

We next hypothesized that AlsSD might play an active and crucial role in detoxifying intracellular acidification that accrues from the deprotonation of weak organic acids in the relatively neutral bacterial cytoplasm [21]. In such a scenario, intracellular protons would be consumed during multiple stages of decarboxylation, first of pyruvate into acetolactate catalyzed by AlsS, followed by that of the acetolactate intermediate into acetoin by AlsD, both leading to a gradual alkalization of the cytoplasm during weak acid stress. Direct evidence confirming a role for AlsSD in regulating intracellular pH was obtained by using cells loaded with the fluorescent pH probe 5 (and 6-)-carboxyfluorescein succinimidyl ester (cFSE). Resting cells that were suspended in potassium phosphate buffer (pH 4.5) maintained a slightly acidic interior (pH_internal_ of 5.9; Fig. 4B, *top*), resulting in a transmembrane pH gradient (ΔpH = pH_internal_- pH_external_) of approximately 1.4 units. Addition of pyruvate under these conditions initiated a pH gradient (ΔpH) decay across the membrane that was exacerbated in the Δ*alsSD* and Δ*cidC* Δ*alsSD* backgrounds compared to either the parental or Δ*cidC* strains (Fig. 4B, *bottom*). Although the pH gradient decay recovered and stabilized over time, the rate and magnitude of the pH_i_ recovery in different strains appeared to be dependent on the activity of AlsSD (Fig. 4B). In the presence of pyruvate, both the parental control and the Δ*cidC* mutant displayed comparable recovery rates of (23.83±1.9)×10^−3^ min^−1^ and (27.45±5.5)×10^−3^ min^−1^, respectively, and reached a pH_i_ comparable to those of control cells (untreated resting cells) within 20 minutes (Fig. 4B, 4C). In contrast, the pH_i_ of both the Δ*alsSD* and Δ*cidC*Δ*alsSD* mutants had stabilized approximately 0.2–0.3 units below that of the pyruvate treated parental control and exhibited significantly decreased pH_i_ recovery rates (*P*<0.05) of (11.66±2.1)×10^−3^ min^−1^ and (5.24±3.8)×10^−3^ min^−1^, respectively leading to incomplete recovery from acidic stress even after 60 minutes (Fig. 4B, 4C). These data demonstrate a role for the enzymatic activity of AlsSD in countering weak acid mediated intracellular acidification of the bacterial cytoplasm.

### Acetate and ROS synergistically promote cell death

In eukaryotes, there is increasing evidence that ROS plays a key role in mediating PCD [22], [23]. Given that the physiological induction of ROS in *S. aureus* is dependent on the accrual of extracellular acetate, we next asked whether cell death is a direct consequence of acetic acid-mediated intracellular acidification or is an indirect result of oxidative stress. To this end we devised a strategy to determine the contribution of intracellular acidification on triggering cell death, independent of the ROS generated after 72 h of growth under aerobic conditions.

**Figure 5:**
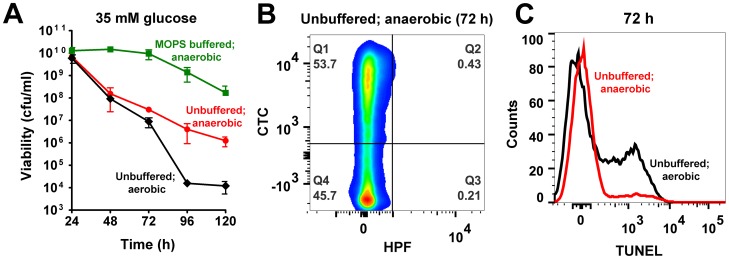
Acetate and ROS contribute towards cell death. (**A**) Aerobically grown cultures of *S. aureus* UAMS-1 (TSB-35 mM glucose or TSB-35 mM glucose 50 mM MOPS, pH 7.3) were shifted to an anaerobic chamber after 24 h of growth. Cell viability was determined daily by counting colonies (cfu/ml) on solid media incubated anaerobically at 37°C. Culture aliquots were sampled 48 h post-shift to anaerobiosis and double stained with HPF/CTC (**B**) and subjected to a TUNEL assay (**C**).

Wild-type *S. aureus* was aerobically grown for 24 h in TSB-35 mM glucose, followed by a sudden shift to anaerobic conditions. While this process ensured as much acidification as aerobically grown cultures, it conveniently eliminated ROS (due to the absence of oxygen). Although it is plausible that a sudden transition of cultures to anaerobic conditions may induce cell death independent of acidic stress, we controlled for this possibility by performing a similar experiment with wild-type *S. aureus* grown in TSB-35 mM glucose buffered to a pH of 7.3 with 50 mM MOPS. Cell viabilities monitored over a 120 h period showed only minimal loss of viability for neutrally buffered cultures, suggesting that cell death following anaerobic transition was dependent on culture acidification, similar to aerobically grown cells (Fig. 5A). Most importantly, unbuffered cultures that were shifted to anaerobic conditions displayed a partial restoration of viability compared to their corresponding aerobic cultures (Fig. 5A). Similarly a partial rescue was also observed when well-aerated cultures grown for 24 h were left standing without further agitation to minimize aeration (Fig. S6). Together, these data suggest a contributory role for oxidative stress in cell death.

Consistent with trends in cell viability, HPF-CTC staining of 72 h unbuffered cultures confirmed the absence of ROS following transition to anaerobiosis and the presence of a respiring population in contrast to that observed for the same time period under aerobic conditions (compare Fig. 5B with Fig. 1B, Table S1). Surprisingly, there was also a dramatic reduction of TUNEL positive cells following the shift to anaerobiosis (Fig. 5C), suggesting that ROS rather than intracellular acidification played a crucial role in DNA damage.

### Staphylococcal cell death affects biofilm development and pathogenesis

**Figure 6:**
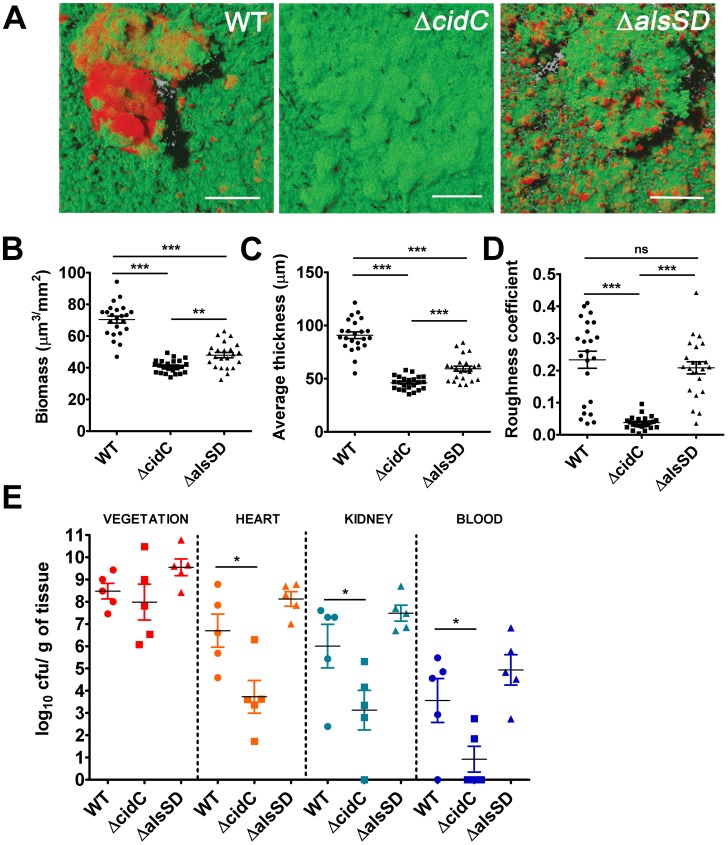
Staphylococcal cell death affects biofilm development and pathogenesis. (**A**) Representative CLSM micrographs of 1-day old biofilms of *S. aureus* UAMS-1 and isogenic mutants stained with SYTO9 (1.3 µM) and TOTO-3 (2 µM) to differentiate between live and dead cells. The images display top-down florescence projections of biofilms grown on glass slides (scale bar: 50 µm). Biofilm biomass (**B**), average thickness (**C**) and roughness coefficients (**D**) were calculated using the COMSTAT image analysis software, (statistical significance was assessed using one way ANOVA followed by Newman Kewl's multiple post-comparison test, n = 23; ** *P*<0.005, *** *P*<0.0005). (**E**) Experimental endocarditis was induced in rabbits by placement of a catheter into the left ventricle (see materials and methods). Bacteria (10^5^ cfu/ml) were inoculated 24 h after catheterization via the marginal ear vein. Animals were euthanized 48 h post infection, organs harvested and bacterial burdens determined by plating on tryptic soy agar plates (*, *P*<0.05, one way ANOVA with Dunnets multiple comparison test, n = 5/strain).

Cell death in staphylococcal biofilms is often spatially restricted to developing microcolonies [24]. Given that the CidR regulon is also actively expressed in microcolonies [24], we predicted that cell death may be modulated by both CidC and AlsSD activities and further contribute to the structural and developmental integrity of the maturing biofilm. To test these hypotheses, we assayed the ability of the Δ*cidC* and Δ*alsSD* mutants to form biofilms, relative to the wild-type strain on glass surfaces exposed to a continuous flow of nutrients. Wild type biofilms appeared as a confluent biomass frequently interspersed with microcolonies that differentiated from the primary biofilm mat. As previously noted, live/dead cell staining of wild-type biofilms confirmed that dead cell populations were predominantly localized within microcolonies (Fig. 6A). However compared to the wild-type strain, COMSTAT analysis of Δ*cidC* biofilms revealed significantly decreased total biofilm biomass and thickness, indicative of developmental defects during biofilm formation (Fig. 6B, 6C). Additionally, the roughness coefficients (a measure of the biofilm architectural heterogeneity) of the Δ*cidC* mutant biofilms were significantly lower than those of wild-type (Fig. 6D), a phenotype that was also consistent with the decreased ability of the Δ*cidC* mutant to differentiate into microcolonies. Finally, the Δ*cidC* biofilm revealed significantly less dead cell biomass compared to the parental strain, strongly suggestive of its involvement in promoting cell death in biofilms (Fig. S7). Similar to the Δ*cidC* biofilm, COMSTAT analysis of biofilms formed by the *ΔalsSD* mutant also exhibited decreased biofilm biomass and thickness compared to the wild-type strain (Fig. 6B, 6C). However it is unlikely that the observed decrease in biomass of one-day old Δ*alsSD* biofilms was due to an early developmental defect as they were able to differentiate into microcolonies and attain similar roughness coefficients to that of its isogenic wild-type strain (Fig. 6D). Rather, these defects are consistent with increased sensitivity of the AlsSD mutant to weak acids and low pH environments of biofilm microcolonies. Collectively, these observations suggest that the activities of both CidC and AlsSD regulate cell death at the population level to achieve optimal biomass and structural integrity during biofilm development.

Since the ability of bacteria to develop as biofilms on heart valves constitutes the root cause of infective endocarditis, we speculated that staphylococcal cell death may also contribute towards pathogenesis in a rabbit model of infective endocarditis. This model not only provides an estimate of the organism's biofilm forming capability *in vivo* but also allows for the simultaneous assessment of embolization (dissemination of the bacterial vegetation to secondary sites due to blood flow associated shear forces in the heart). To test this hypothesis we induced left-sided endocarditis in rabbits and infected them with wild-type, Δ*cidC* and Δ*alsSD* mutant strains. At 48 h post-infection, the bacterial burden in the primary vegetation (heart valves), heart tissue, kidney and blood were determined. Consistent with being the primary site of biofilm infection, the heart valves exhibited the maximum bacterial burden (∼10^8^ cfu/gm of tissue) among the various tissues harvested (Fig. 6E). Relative to the wild-type strain, both Δ*cidC* and Δ*alsSD* mutants had similar bacterial loads at this site (Fig. 6E) suggesting that these metabolic pathways did not affect the growth of these strains or their ability to colonize the primary infection sites *in vivo*. Interestingly however, the Δ*cidC* mutant displayed significantly decreased bacterial burdens in the blood and other secondary sites of infection including heart tissue (excluding valves) and kidneys (Fig. 6E). As bacterial colonization of these secondary sites primarily results from infectious emboli originating from the heart valve, it may be argued that the cell-death associated CidC pathway may play a role in metastasis of the valvular vegetation.

## Discussion

In the present study, we demonstrate that both CidC and AlsSD pathways, that have traditionally been considered metabolic routes for excess carbon flow, antithetically modulate staphylococcal cell death by regulating the levels of excreted acetic acid. Exercising such metabolic control over cell death affords *S. aureus* a means to modulate biofilm development and possibly disperse and colonize alternate sites during the course of biofilm-associated infections.

How does acetate potentiate cell death? Our results reveal that both intracellular acidification and ROS generation may play a role in acetate dependent cell death. Although intracellular acidification can result from other fermentative metabolites like D- or L-lactate, we were unable to detect excretion of L-lactate and observed only small differences in the excretion of D-lactate in the Δ*cidC* and Δ*alsSD* mutants relative to WT (Fig. S8). Furthermore, given that the pK*a* of D-lactate (pK*a* = 3.86) is lower than acetate and its levels in culture supernatants were minute (∼35 fold less than acetate) we reasoned that it is unlikely to have a similar effect to that of acetic acid on cell death during aerobic growth. At the molecular level, it is not clear how acetate initiates ROS production. The evidence presented in this study suggests that acetate may contribute to a bottle-neck in electron transport by reducing the functionality of the respiratory chain. This could catalyze the promiscuous reduction of molecular oxygen and result in the production of ROS. Consistent with this argument, we have confirmed increased levels of superoxide and hydroxyl radicals following growth of *S. aureus* in 35 mM glucose, a condition that leads to acetate stress. Given that the pK*a* of superoxide anion is 4.88, it is very likely that this species freely traverses the cytoplasmic membrane and mediates oxidative damage during acetic acid stress. In addition to ROS, cytoplasmic acidification due to acetate influx itself can be a significant cause of cell death. Evidence for this conclusion arises from the observation that both metabolizable (acetate, lactate and pyruvate) and nonmetabolizable (benzoate) weak acids inhibit *S. aureus* growth. Such inhibition can result from acid catalyzed intracellular protein unfolding and aggregation. In support of this conclusion, a recent transcriptomic analysis of *S. aureus* challenged with the weak acid, lactate, revealed various *clp* genes (including *clpB*, *clpC* and *clpP*) involved in protein folding and recycling to be strongly up regulated [25].

Extending these findings to biofilms, we argue that the acidic pH microenvironments of biofilm microcolonies [26], [27] may spatially bias these biological structures as sites of respiratory inhibition and cell death. Weak acid metabolic byproducts like acetate and lactate are thought to accumulate within the biofilm interior, primarily due to diffusion limits resulting from reduced fluid flow and accumulation of biofilm matrix components [28]. We propose a model of staphylococcal PCD wherein the acidic pH within microcolonies activates expression of the CidR regulon [29] in a subpopulation of cells. This subsequently would lead to a feed-forward loop in which toxic acetate levels are reached through CidC activity. Ultimately cell death would ensue when macromolecular repair mechanisms are exhausted and cells within the biofilm are overwhelmed by the damaging effects of acetate (Fig. S9). To prevent a disproportionate number of cells from undergoing cell death, *S. aureus* co-expresses the AlsSD pathway along with CidC. Enzymatic activity of acetolactate synthase (AlsS) results in the condensation of two molecules of pyruvate to acetolactate and thereafter to acetoin by acetolactatae decarboxylase (AlsD). This effectively minimizes the carbon diverted to the generation of toxic acetate through the CidC pathway. More importantly evidence presented here also shows that the AlsSD pathway consumes protons from the cytoplasm and helps maintain pH homeostasis similar to the enterococci and lactobacilli [21], [30]. We contend that the ability of AlsSD to antagonize CidC activity effectively results in the modulation of intracellular pH and constitutes a robust mechanism to limit cell death and optimize biomass within the microenvironment of a microcolony.

The pathways that generate acetate vary among organisms. Whereas most bacteria, including *E. coli* and *S. aureus* generate acetate under aerobic conditions through the Pta-AckA and the CidC pathways, most eukaryotes (yeasts and mammals) lack these enzymes. Instead yeasts produce acetate as a natural byproduct of ethanol fermentation from acetaldehyde using acetaldehyde dehydrogenase [31]. Mammalian cells rarely generate significant quantities of acetate. But under certain conditions, acetate is produced by the enzymatic hydrolysis of acetyl-CoA in the cytoplasm [32]. Irrespective of the diversity in production routes, multiple studies have demonstrated that acetate itself can act as a potent inducer of PCD in yeasts and mammalian cells [33], [34]. Consistent with these studies, we demonstrate multiple hallmarks of eukaryotic PCD including respiratory dysfunction, generation of ROS and DNA fragmentation to be conserved during acetate mediated cell death in *S. aureus*. Further, similar to eukaryotic PCD [1], acetate-mediated cell death functions in a developmental context and appears to be crucial for optimal staphylococcal biofilm development.

It is noteworthy that two different activities of pyruvate oxidase (CidC, also annotated as PoxB in *E. coli* and SpxB in *S. pneumoniae*) have been described previously [35], [36]. In *E. coli* and *S. aureus*, this enzyme catalyses the decarboxylation of pyruvate into acetic acid and carbon dioxide, whereas acetyl phosphate and hydrogen peroxide are the predominant products of a similar reaction in *L. plantarum* and *S. pneumoniae*. Remarkably, both these reactions appear to induce stationary phase cell death in bacteria with either acetate or hydrogen peroxide as principal determinants of cell death [37]. Additionally, similar to *S. aureus*, cell death due to pyruvate oxidase activity is associated with apoptotic hallmarks in *S. pneumoniae* [37]. These observations appear to clearly mark pyruvate oxidase activity as a suicidal marker in bacteria.

Finally, what are the biological implications of regulating PCD? We used a well-established rabbit model of infective endocarditis to assess the effects of altering PCD on *in vivo* biofilm development. *S. aureus* injected intravenously in rabbits is rapidly cleared from the blood within the first 30 minutes leaving only minute residual amounts to linger over longer periods of time [38]. However any injury to the heart valves marks a preferred site for bacterial colonization and eventual development into a biofilm (vegetation). The pathological progression of infective endocarditis subsequently involves embolization of bacterial vegetations to alternate sites including surrounding heart tissues and other peripheral organs like the brain and kidneys [39]. This process not only poses a constant seeding source of infection but also hinders the normal functioning of peripheral organs and is often associated with a high degree of mortality [39]. Our investigations failed to reveal a colonization defect of the Δ*cidC* and Δ*alsSD* mutants on heart valves relative to the wild-type strain. However we observed a significant decrease in Δ*cidC* burdens in the blood, heart and kidneys after 48 h of infection. Although not conclusive, these findings strongly suggest that the Δ*cidC* mutant had lower rates of dissemination to secondary infection sites *in vivo*. Alternately, it is also possible that the Δ*cidC* mutant exhibits tissue specific fitness and survival defects *in vivo*. Either way, these findings suggest that alterations to the activity of cell-death associated metabolic pathways during biofilm development could affect staphylococcal pathogenesis.

In conclusion, the activation of pathways that generate metabolic acids from glucose during carbon-overflow and oxygen replete conditions have long been considered paradoxical in bacteria that are capable of undergoing oxidative phosphorylation, as it results in low energy yields, potentially toxic acid by-products and activation of cell death pathways [40]. Based on the current study we propose that the extracellular accumulation of metabolic acids is a developmental strategy that bacteria undertake to initiate cell death, a necessary precursor to optimal biofilm development. The initiation of staphylococcal cell death by intracellular acidification bears some striking resemblance to eukaryotic PCD. For instance, the dimerization and insertion of the pro-apoptotic modulator, Bax, into the membrane is thought to be triggered by intracellular acidification of eukaryotic cells [41] just prior to the release of cytochrome c into the cytoplasm. In this regard, it is possible that membrane oligomerization of CidAB and LrgAB (functional analogs of Bax and Bcl-2 in *S. aureus*, respectively) may also be initiated following intracellular acidification. Additionally, cytoplasmic acidification in eukaryotes also activates caspases, essential components of the apoptotic pathway [42]. Collectively, these observations are suggestive of a conserved role for glycolysis-mediated intracellular pH regulation in the modulation of PCD in eukaryotes and prokaryotes.

## Materials and Methods

### Ethics

Animal experiments were conducted in compliance with a protocol (# 12-048-08-FC) approved by the Institutional Animal Care and Use Committee (IACUC). The University of Nebraska Medical Center is accredited by the Association of for Assessment and Accreditation of Laboratory Animal Care International (AALAC). In addition, all animals at the University of Nebraska Medical Center are maintained in accordance with the Animal Welfare Act and the DHHS “Guide for the Care and Use of Laboratory Animals.”

### Bacterial strains, plasmids and growth conditions

Strains and plasmids used in this study are listed in Table S2. The Δ*cidC*Δ*alsSD* double mutant was created by moving the Δ*cidC*::*erm* allele from KB1058 into the Δ*alsSD* mutant (UAMS-1489) using bacteriophage Φ11-mediated transduction. In addition to growth on selective antibiotic media, the Δ*cidC* transductants were confirmed phenotypically and by PCR using the following primer pairs: cidC UP (5′-CACATGCATTTGGCACAGCT-3′) and cidC DN (5′-TGCTCATGCCTGCATTACCA-3′). The plasmid, pVCT2, containing the *alsS* gene with its native promoter was constructed by amplifying an approximately 2-kb region from the UAMS-1 genome using the primers, *alsS*-comp-F (5′-GATCGAGCTCTCCCTTATAATCACTCCCTTCA-3′) and *alsS*-comp-R (5′-AGTCTCTAGATGTGCCTAATGTACCATGTTG-3′), and inserting the resulting DNA fragment into the Sac1 and Xba1 sites of the shuttle vector, pLI50 [43]. Similarly, plasmid pVCT3 (containing *cidC* gene with its native promoter) was amplified from a Δ*cidAB* double mutant using primers, *cidC* comp-F (5′-GATCGAATTCACTCATTATTTGTGATTCCTCA-3′) and *cidC* comp-R (5′-AGTCGTCGACCAATTCAGTACAATCATTTGTG-3′). The resulting amplification product was inserted into the EcoR1 and Sal1 sites of pLI50. Subsequently, both pVCT2 and pVCT3 were transformed into RN4220 and transduced into the Δ*alsSD* and Δ*cidC* mutants respectively, using bacteriophage ϕ11 for phenotypic complementation.

*E. coli* cultures were grown in Luria Bertani (LB) broth. *S. aureus* cultures were grown in trypticase soy broth (TSB) supplemented with 35 mM glucose (unless specified otherwise). Bacterial cultures were aerobically grown at 37°C in either Erlenmeyer flasks fitted with bug stoppers to minimize evaporation during long-term growth or in 96-well flat, clear bottom micro-titer plates. For anaerobic growth, cultures were supplemented with cysteine (0.5 mg/ml) and 10 mM nitrate (or fumarate) and agitated at 250 rpm in an anaerobic hut. When necessary, antibiotics were added to cultures as follows: ampicillin (100 µg/ml); erythromycin (5 µg/ml); tetracycline (10 µg/ml); and chloramphenicol (10 µg/ml).

### Flow cytometry

All analyses were performed using 1- and 3- day old stationary phase cultures of *S. aureus* on a BD LSRII flow cytometer (Beckton and Dickinson, San Jose, California). Cell samples were washed twice and diluted to a final concentration of 10^7^ cells per ml in PBS. Cells were stained for 30 min with 5-cyano-2,3-ditolyl tetrazolium chloride (CTC, 5 mM) and 3-(p-hydroxyphenyl) fluorescein (HPF, 15 µM) followed by FACS analyses at a flow rate of ∼1000 cells per second. A total of 10000 events were collected for each sample. Bacteria were discriminated from background using a combination of forward scattered ligt (FSC) and side scattered light (SSC). Samples were excited at 488 nm using an argon laser and HPF emission was detected at 530±30 nm (with a 505 nm long-pass mirror) whereas CTC emission was detected at 695±40 nm (with a 685 nm long-pass mirror). Raw data were analyzed using the FlowJo software.

### TUNEL assay

Quantitative assessment of DNA fragmentation was performed using the ApoDirect kit (BD bioscience). Samples were collected at the appropriate time points and fixed in 1% paraformaldehyde for 30 minutes. Cells were then washed twice in PBS, resuspended in 70% ethanol and stored at −20°C. Subsequent labeling of 3-OH ends of fragmented DNA was performed according to the manufacturer's instructions. Flow cytometry to detect TUNEL positive cells was performed as previously described [4].

### Electron paramagnetic resonance (EPR) spectroscopy

Aliquots from stationary phase cultures (1- and 3 days) were withdrawn and resuspended to an OD_600_ of 10 units in 1 ml KDD buffer (Krebs-HEPES buffer, pH 7.4; 99 mM NaCl, 4.69 mM KCl, 2.5 mM CaCl_2_, 1.2 mM MgSO_4_, 25 mM NaHCO_3_, 1.03 mM KH_2_PO4, 5.6 mM D-glucose, 20 mM HEPES, 5 µM DETC and 25 µM deferoxamine). The resuspended culture aliquots were then incubated with 200 µM of cell-permeable ROS sensitive spin probe 1-hydroxy-3-methoxycarbonyl-2,2,5,5-tetramethylpyrrolidine (CMH; Noxygen Science Transfer and Diagnostics, Elzach, Germany) for 15 minutes at room temperature prior to analysis using a Bruker e-scan EPR spectrometer with the following settings: field sweep width, 60.0 gauss; microwave frequency, 9.75 kHz; microwave power, 21.90 mW; modulation amplitude, 2.37 gauss; conversion time, 10.24 ms; time constant, 40.96 ms. To identify the nature of ROS produced, cells resuspended in KDD buffer were incubated with either 400 units of superoxide dismutase (SOD; O_2_^•−^ scavenger) or cell permeable dimethyl thiourea (20 mM DMTU; OH^•^ scavenger) prior to the addition of CMH.

### Oxygen consumption

*S. aureus* was cultured at 37°C in TSB supplemented with either 14 or 35 mM glucose and aerated at 250 rpm with a flask-to-medium ratio of 10∶1 for 24 h. Cultures were subsequently diluted to an OD_600_ of 0.1 in fresh TSB (14 mM glucose). Oxygen consumption rates were determined for a period of 30 minutes at 37°C by using a MitoXpress oxygen-sensitive probe (Luxcel Biosciences) according to the manufacturer's instructions. The data were normalized to the corresponding OD_600_ units.

### Metabolite analyses

For these analyses, bacterial growth was allowed to proceed at 37°C and 200 rpm in BugStopper-sealed flasks containing TSB (35 mM glucose) in a 1∶10, flask to volume ratio. Preliminary experiments suggested that the assayed metabolite by-products were not significantly consumed following exhaustion of glucose from the media for up to 24 h. Therefore metabolite excretion profiles were determined from culture supernatants that were harvested at 24 h of growth. Acetate and glucose from culture supernatants were measured using commercial kits (R-Biopharm, Marshall, MI), according to the manufacturer's instructions. Acetoin was measured as previously described [44].

### Measurement of bacterial growth

Overnight grown (16 to 18-hr) *S. aureus* cultures were resuspended to an OD_600_ of 0.06 in TSB (35 mM glucose). Bacterial suspensions were dispensed into 96-well microtiter plates and grown for 24 h at 37°C in a Tecan infinite 200 spectrophotometer under maximum aeration. The absorbance signals (OD_600_) were recorded every 30 minutes for the entire period of growth. For various experiments bacteria were challenged with the following compounds (final concentrations): acetic acid (30 mM), lactic acid (40 mM), pyruvic acid (30 mM) and acetoin (10 mM).

### Intracellular pH determination

Intracellular pH was determined as previously described [30] with the minor modifications. Briefly, bacterial cells were grown in TSB (35 mM glucose) and 1 mL was harvested by centrifugation upon reaching an OD_600_ of 2. Cells were washed twice with an equal volume of 10 mM potassium phosphate buffer (pH 7) and resuspended in an equal volume of the same buffer. To load cells with the intracellular pH probe, 10 µl of 1 mM CFDA SE (5-(and 6)- carboxyfluorescein diacetate succinimidyl ester) was added to the suspension and incubated for 15 minutes at 30°C. Excess dye was removed by incubating the cells for 15 minutes at 30°C in potassium phosphate buffer (pH 7) containing 10 mM glucose. Cells were subsequently washed twice in the same buffer and finally resuspended in 50 mM potassium phosphate buffer (pH 4.5). Labeled cells were kept on ice until use.

To measure intracellular pH, 100 µl of the labeled cell suspensions were introduced into a 96-well flat bottom, black polystyrene plate (COSTAR 3916). Fluorescence was measured using a Tecan Infinite 200 spectrofluorimeter with excitation and emission wavelength set at 490 nm and 525 nm, respectively. Fluorescence emission units were converted to pH units using a standard calibration curve derived from labeled cells whose internal pH was equilibrated to the external pH (in citric acid buffers) ranging from pH 4 to 8, by the addition of 1 mM valinomycin and 1 mM nigericin.

### Biofilm assays and confocal microscopy

Biofilms were grown in either FC280 or FC285 flow-cell systems (Biosurfaces Technology Inc, Bozeman, MT) and were analyzed by CLSM as described previously [45]. Briefly, biofilms stained with SYTO-9 (1.3 µM final concentration) and TOTO-3 (2 µM final concentration) fluorophores were excited with the 488 nm and 633 nm lasers respectively, and the emissions were collected using a 525±25 nm and 680±30 nm band-pass filter. For pictorial representation, the biofilms were imaged using an Achroplan 40×0.8 n.a. water dipping objective and for COMSTAT image analysis, images were acquired using a 20×1.2 n.a. dry objective to achieve a larger biofilm surface area for statistical purposes. Regions of interest within the biofilms were selected from similar areas within each flow-cell chamber and each confocal experiment was repeated a minimum of three times. Biofilm architecture was characterized using the COMSTAT software and measures of total biomass, average thickness, maximum height and roughness coefficients were determined [46]. Images were rendered using Imaris software (Bitplane, Saint Paul, MN).

### Experimental endocarditis

Experimental endocarditis on the aortic valve of female New Zealand White rabbits (3 kg) were carried out as previously described with minor modifications [47]. Briefly, rabbits were anesthetized by intramuscular injection of ketamine hydrochloride (35–50 mg/kg), xylazine (2.5–6 mg/kg) and atropine (0.005–0.01 mg/kg) cocktail. An incision was made dextrolateral to the trachea and a polyethylene catheter (Becton Dickinson, MD) was then introduced into the left ventricle via the right carotid artery to produce sterile thrombotic endocarditis, and the skin incision sutured. To induce bacterial endocarditis, animals were intravenously challenged with 1 ml inocula (10^5^ cfu/ml) via the marginal ear vein 24 h after catheterization. The animals were challenged with either the wild-type or mutant strains (Δ*cidC* and Δ*alsSD*) and subsequently (48 h post-infection) euthanized by lethal injection of a solution containing sodium pentobarbital (200 mg/kg). Bacterial loads from various tissue homogenates were determined by serial dilutions on THB agar plates.

## Supporting Information

Figure S1 **Chemical nature of ROS produced during cell death.** ROS detected by EPR was composed of superoxide and hydroxyl radicals based on the quenching of EPR signal by superoxide dismutase (SOD; 400U) and dimethyltiourea (DMTU; 20 mM).
(PDF)

Figure S2 **Cell death is potentiated following growth in diverse carbon sources.** (**A**) *S. aureus* UAMS-1 cell viabilities (cfu/ml, mean ± SD) were monitored every 24 h over a period of five days in TSB containing 45 mM of carbon source (glucose, fructose, sucrose and mannitol). Psuedocolor density plots of cells double stained with HPF and CTC (**B**), whole cell EPR analysis (**C**) and TUNEL staining (**D**) were carried out with cells grown in different carbon sources after 72 h growth (1∶10 flask to volume ratio, 37°C, 250 rpm). The total percent of TUNEL positive cells were calculated based on an unstained control.
(PDF)

Figure S3 **Complementation of Δ** ***cidC*** **and Δ** ***alsSD*** **mutant phenotypes.** (**A**) Cell viabilities (cfu/ml, mean ± SD) of *S. aureus* UAMS-1 (WT pLI50), *cidC* compl. and Δ*alsSD* compl. were monitored every 24 h over a period of five days in TSB-35 mM glucose. Psuedocolor density plots of cells double stained with HPF/CTC (**B**), whole cell EPR analysis (**C**) and TUNEL staining (**D**) were carried out after 24 and 72 h growth.
(PDF)

Figure S4 **Mutation of** ***alsSD*** **renders** ***S. aureus*** **susceptible to weak acids.** Overnight grown (16 to 18-hr) *S. aureus* cultures (WT and Δ*alsSD*) were resuspended to an OD_600_ of 0.06 in (**A**) TSB-35 mM glucose (untreated) or TSB-35 mM glucose supplemented with (**B**) acetic acid (30 mM), (**C**) lactic acid (40 mM) or (**D**) pyruvic acid (30 mM). Bacterial suspensions were dispensed into 96-well microtiter plates and grown for 24 h at 37°C in a Tecan infinite 200 spectrophotometer under maximum aeration. The absorbance signals (OD_600_) were recorded every 30 minutes for the entire period of growth.
(PDF)

Figure S5 **Acetoin does not rescue pyruvic acid mediated growth inhibition of** ***alsSD*** **mutants.** Overnight grown cultures of Δ*alsSD* mutant (**A**) or Δ*cidC* Δ*alsSD* double mutant (**B**) were seeded to a final OD_600_ of 0.06 in TSB-35 mM glucose supplemented with excess acetoin (10 mM). Cultures were challenged with 30 mM pyruvic acid (Py) and growth was monitored for 24 h at 37°C in a Tecan infinite 200 spectrophotometer under maximum aeration.
(PDF)

Figure S6 **Microaerobic growth partially rescues glucose dependent stationary phase cell death.** *S. aureus* UAMS-1 cultures aerobically grown for 24 h at 250 rpm were shifted to static conditions without agitation in a 37°C incubator for a total of 120 h. Cell viability was determined daily.
(PDF)

Figure S7 **COMSTAT analysis of** ***S. aureus*** **UAMS-1 and metabolic mutant biofilms.** (**A**) Maximum thickness of biofilms (**B**) Fold changes in dead cell biomass relative to wild-type biofilm. Fold change was determined after normalization of dead cell biomass to the total biomass (Statistical significance was assessed using one way ANOVA followed by Newman Kewl's multiple post-comparison test; ** *P*<0.005, *** *P*<0.0005).
(PDF)

Figure S8 **D-lactate levels in** ***S. aureus*** **culture supernatants.** The concentration of D-lactate was determined from culture supernatants of various *S. aureus* strains after 24 h of growth in TSB-35 mM glucose using a commercially available kit (R-Biopharm, Germany). Statistical significance was assessed using one way ANOVA followed by Newman Kewl's multiple post-comparison test, n = 3; * *P*<0.05).
(PDF)

Figure S9 **Schematic of the proposed regulation of PCD in** ***S. aureus*** **.** Excess acetate (A^−^) generated by CidC activity contributes to the decrease in external pH within biofilm microcolonies. When the pH_external_ approaches the pK*a* of acetic acid (∼4.8), the undissociated neutral form of the acid (AH) enters the cell resulting in cytoplasmic acidification. This leads to the inhibition of cellular respiration and ROS is generated in the process. Ultimately programmed cell death may result from irreparable damage to biological macromolecules like proteins, RNA and DNA exacted by acid and oxidative stress. Alternately PCD may also result from a weak acid dependent olgomerization and insertion of CidAB and/or LrgAB proteins within the membrane. To counter and limit PCD, cells limit the generation of acetate by re-routing pyruvate to acetoin (neutral) production via the AlsSD pathway. Additionally, the activity of AlsSD also results in the consumption of protons and this helps in maintenance of pH homeostasis.
(PDF)

Table S1 **Flow cytometry- quadrant statistics (%).**
(DOC)

Table S2 **Strains and plasmids.**
(DOC)

Text S1 **Supporting references.**
(DOC)
