# Supplementary figures and images for: A Central Role for Carbon-Overflow Pathways in the Modulation of Bacterial Cell Death
Source: PLoS Pathog. 2014 Jun 19;10(6):e1004205. doi: 10.1371/journal.ppat.1004205 (PMC4063974; doi:10.1371/journal.ppat.1004205)

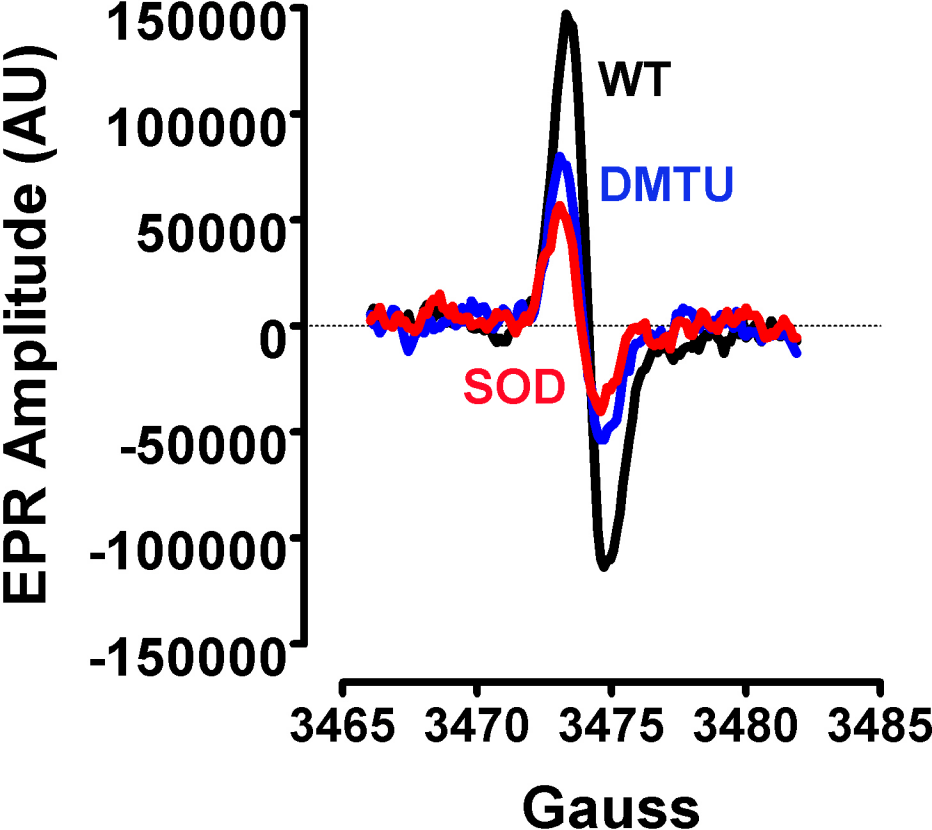

Supplement: Figure S1 — Chemical nature of ROS produced during cell death. ROS detected by EPR was composed of superoxide and hydroxyl radicals based on the quenching of EPR signal by superoxide dismutase (SOD; 400U) and dimethyltiourea (DMTU; 20 mM). (PDF) [file ppat.1004205.s001.pdf]

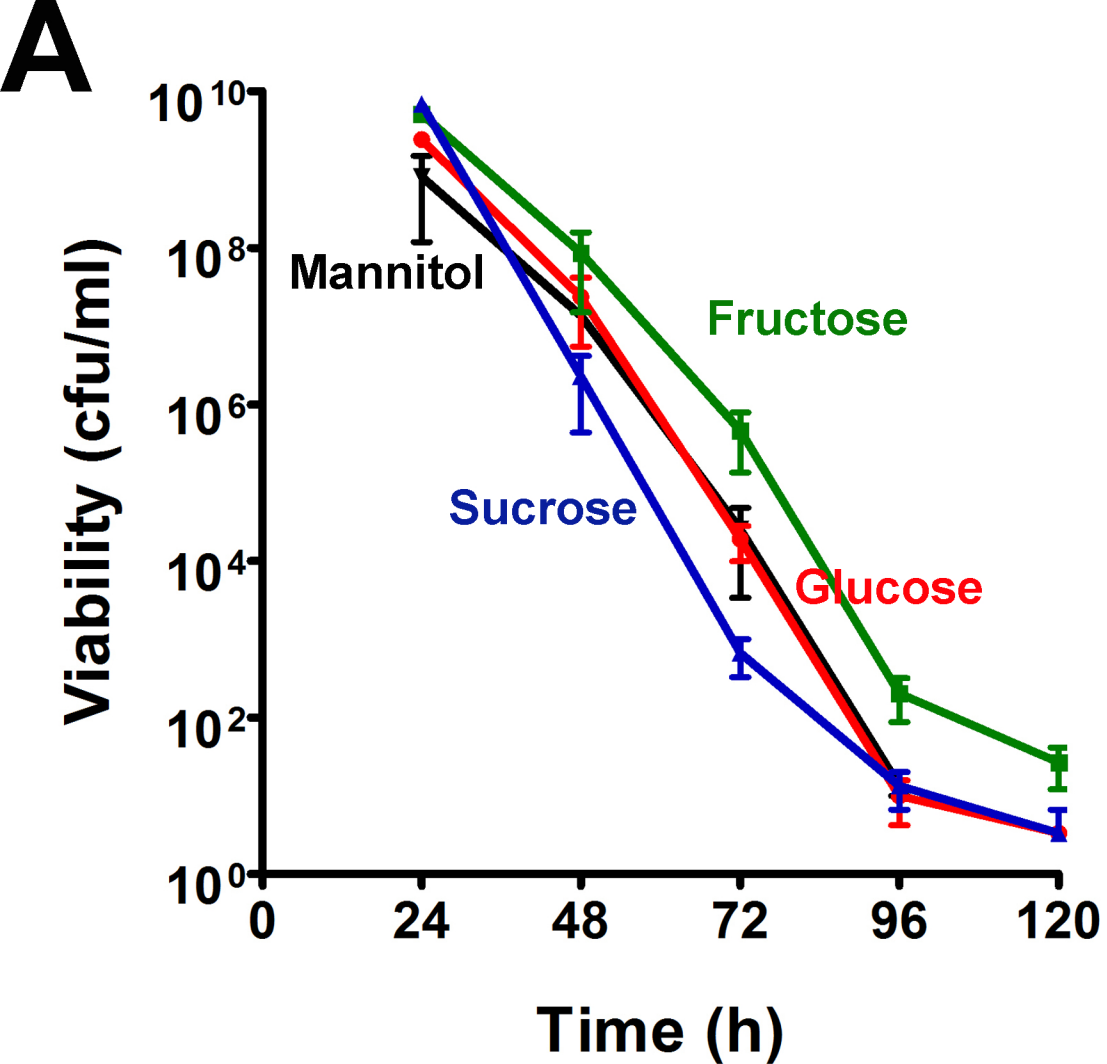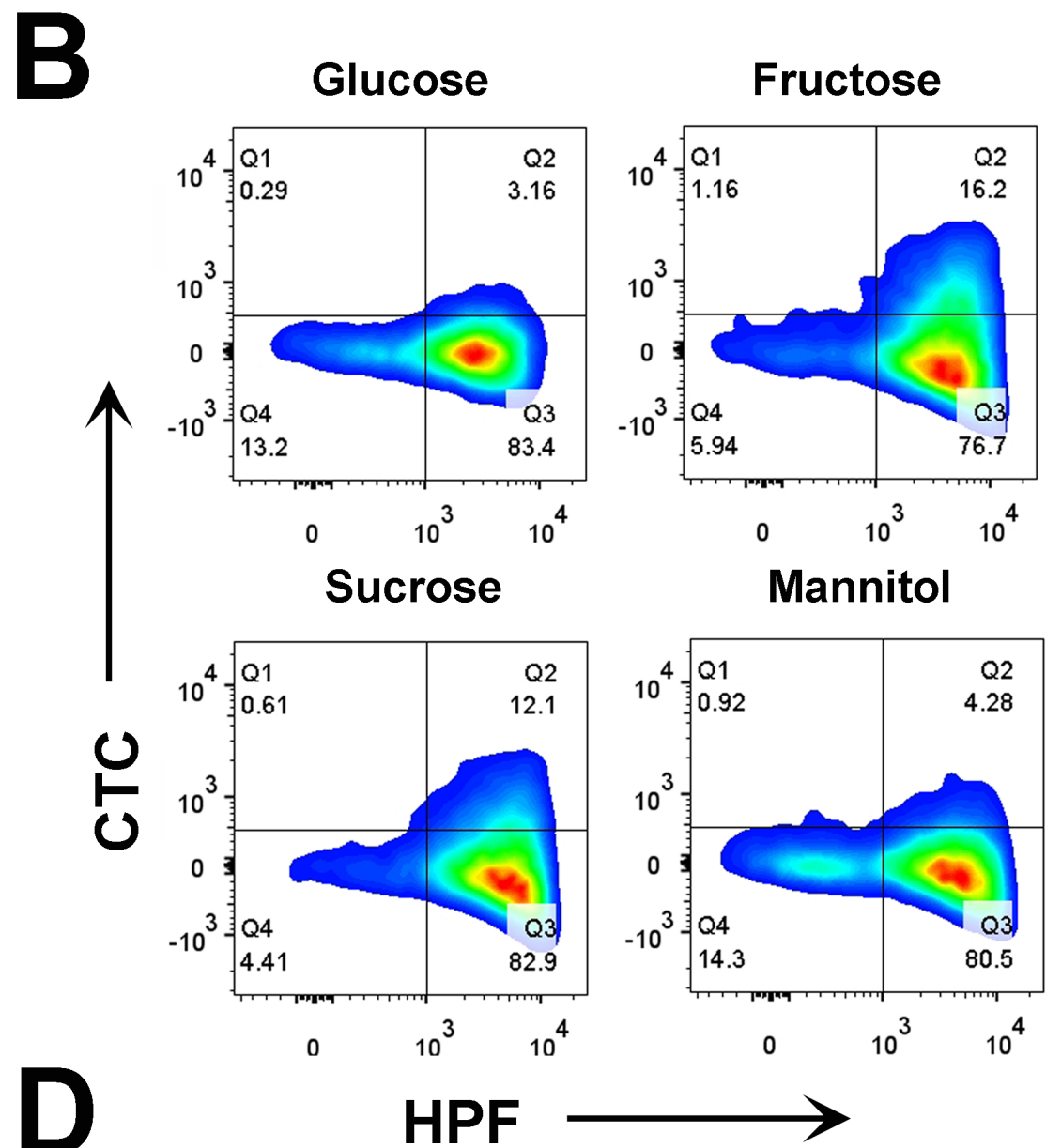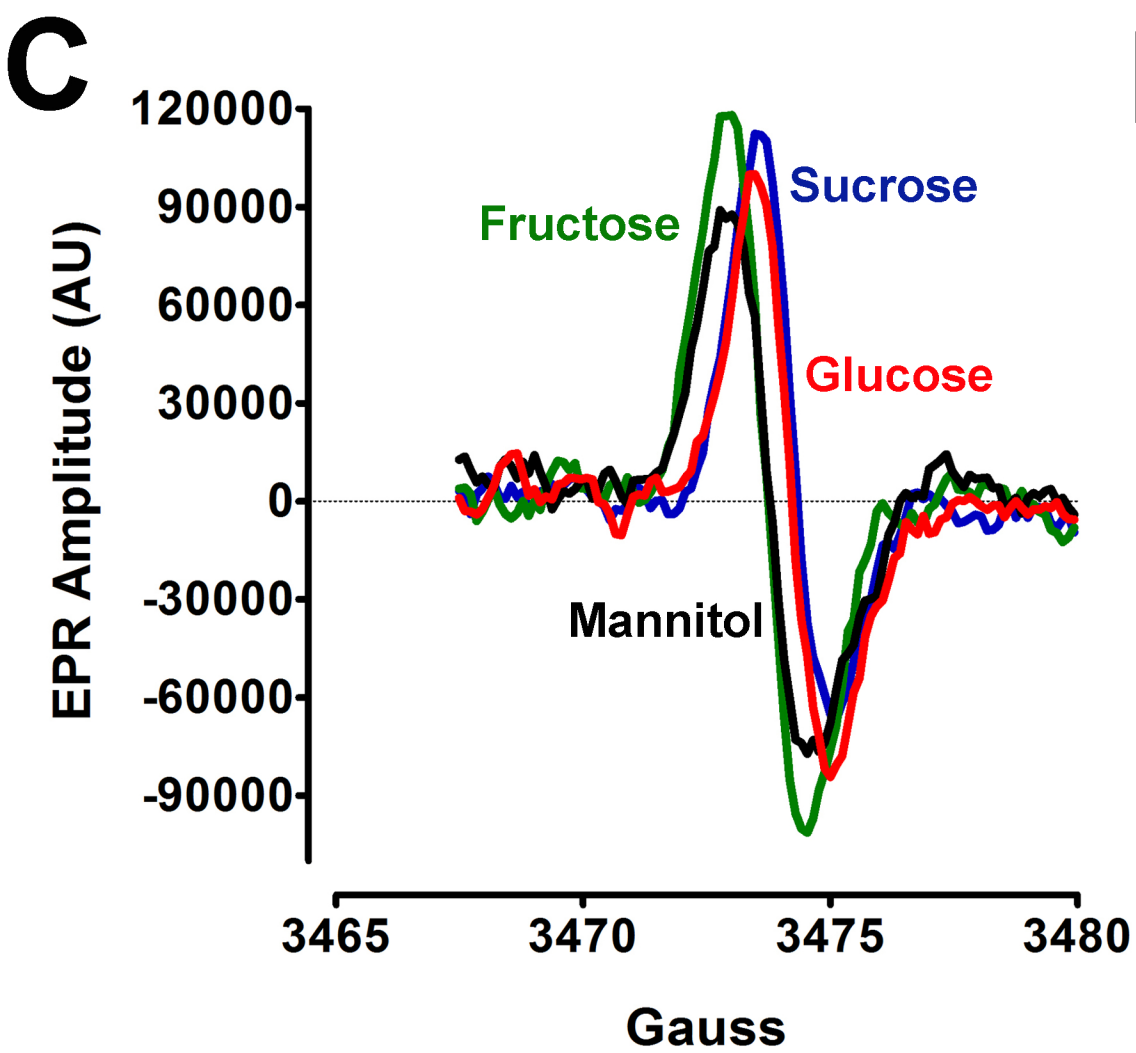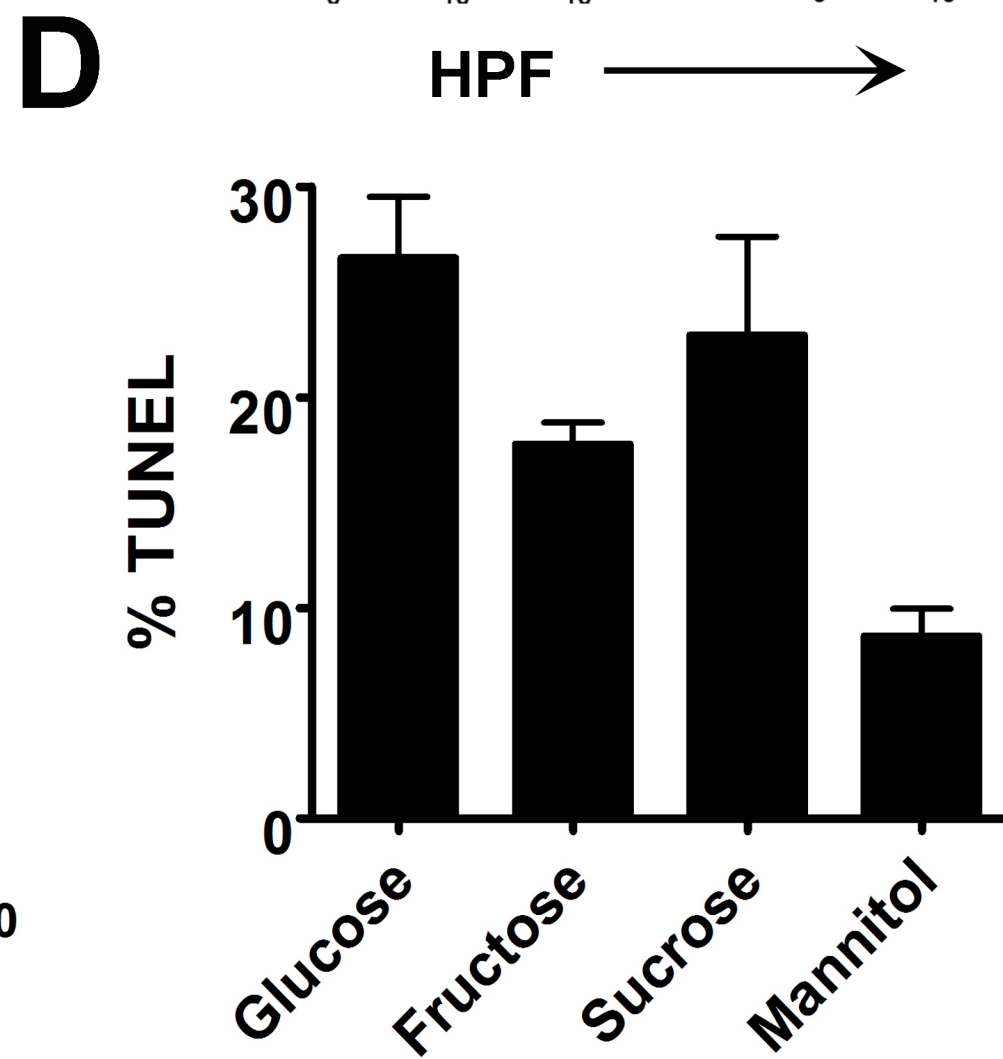

Supplement: Figure S2 — Cell death is potentiated following growth in diverse carbon sources. (A) S. aureus UAMS-1 cell viabilities (cfu/ml, mean ± SD) were monitored every 24 h over a period of five days in TSB containing 45 mM of carbon source (glucose, fructose, sucrose and mannitol). Psuedocolor density plots of cells double stained with HPF and CTC (B), whole cell EPR analysis (C) and TUNEL staining (D) were carried out with cells grown in different carbon sources after 72 h growth (1∶10 flask to volume ratio, 37°C, 250 rpm). The total percent of TUNEL positive cells were calculated based on an unstained control. (PDF) [file ppat.1004205.s002.pdf]

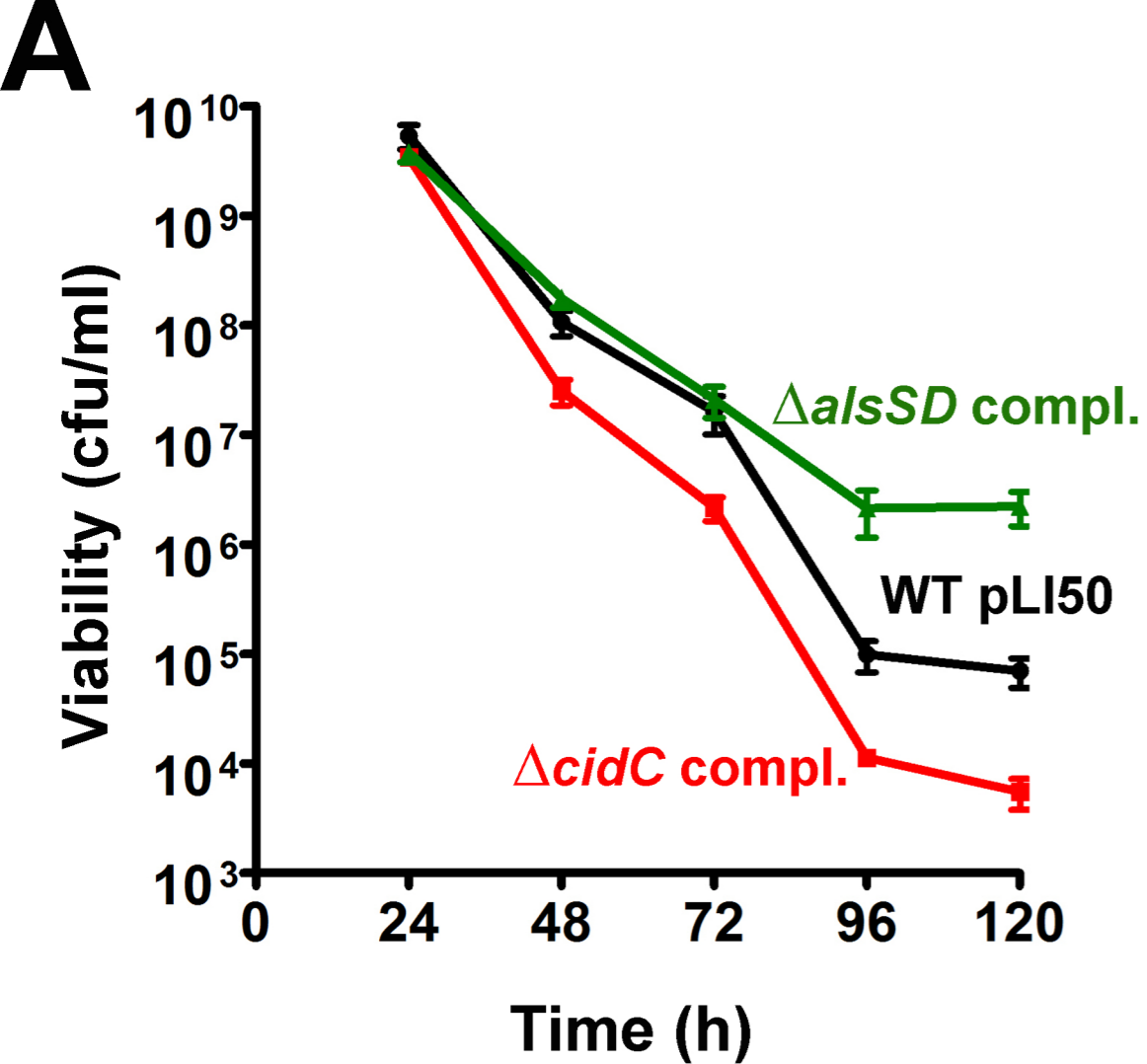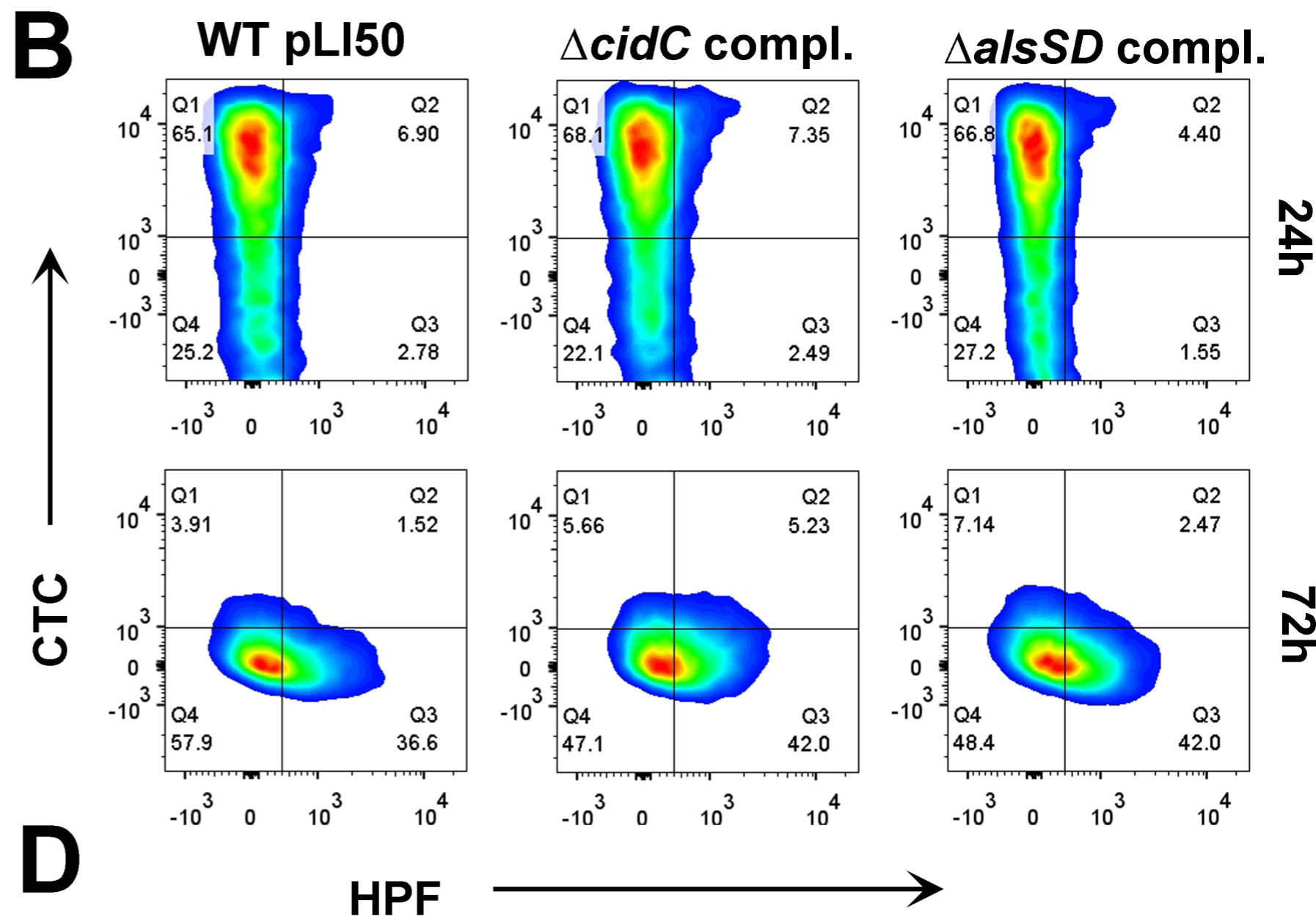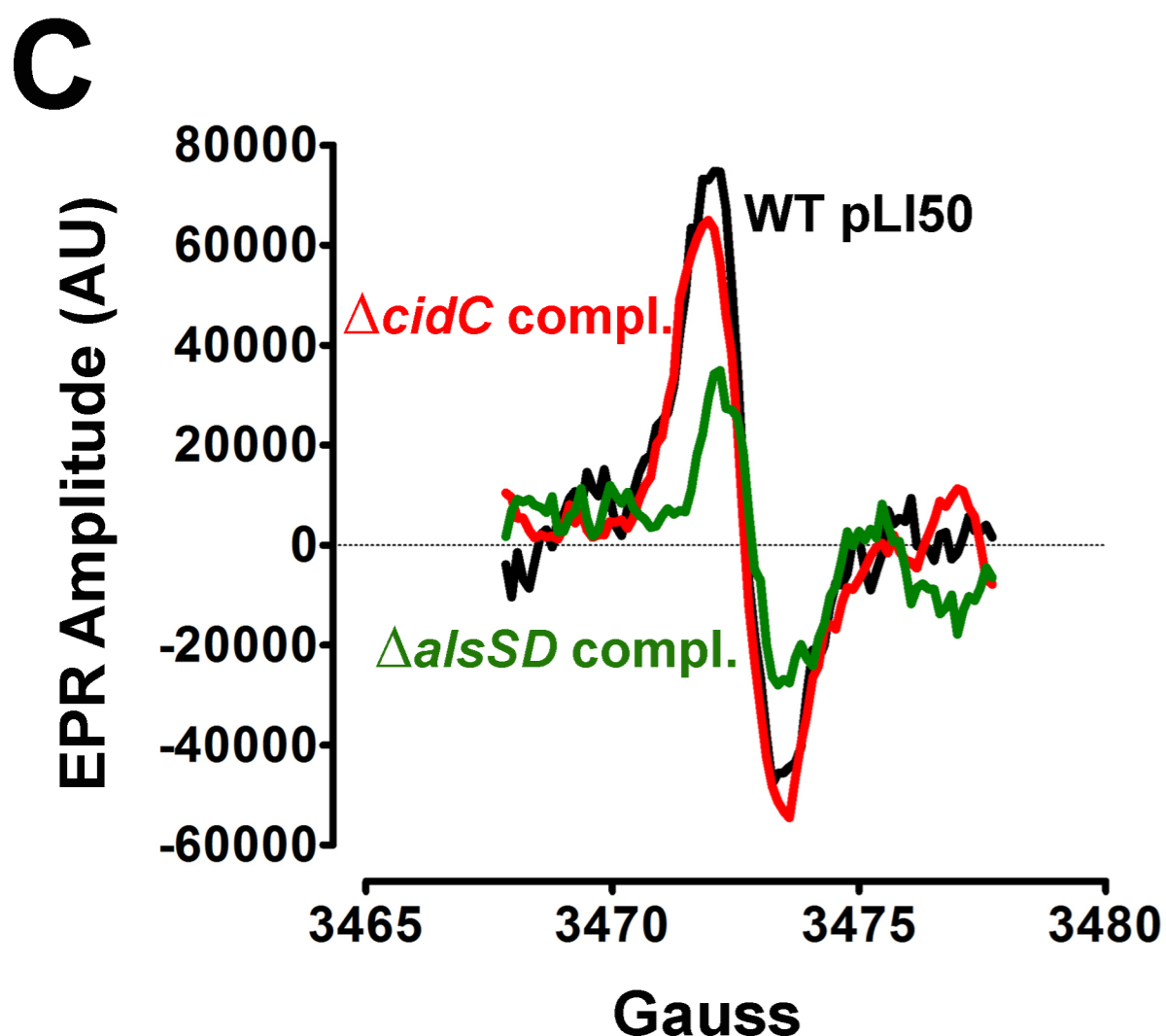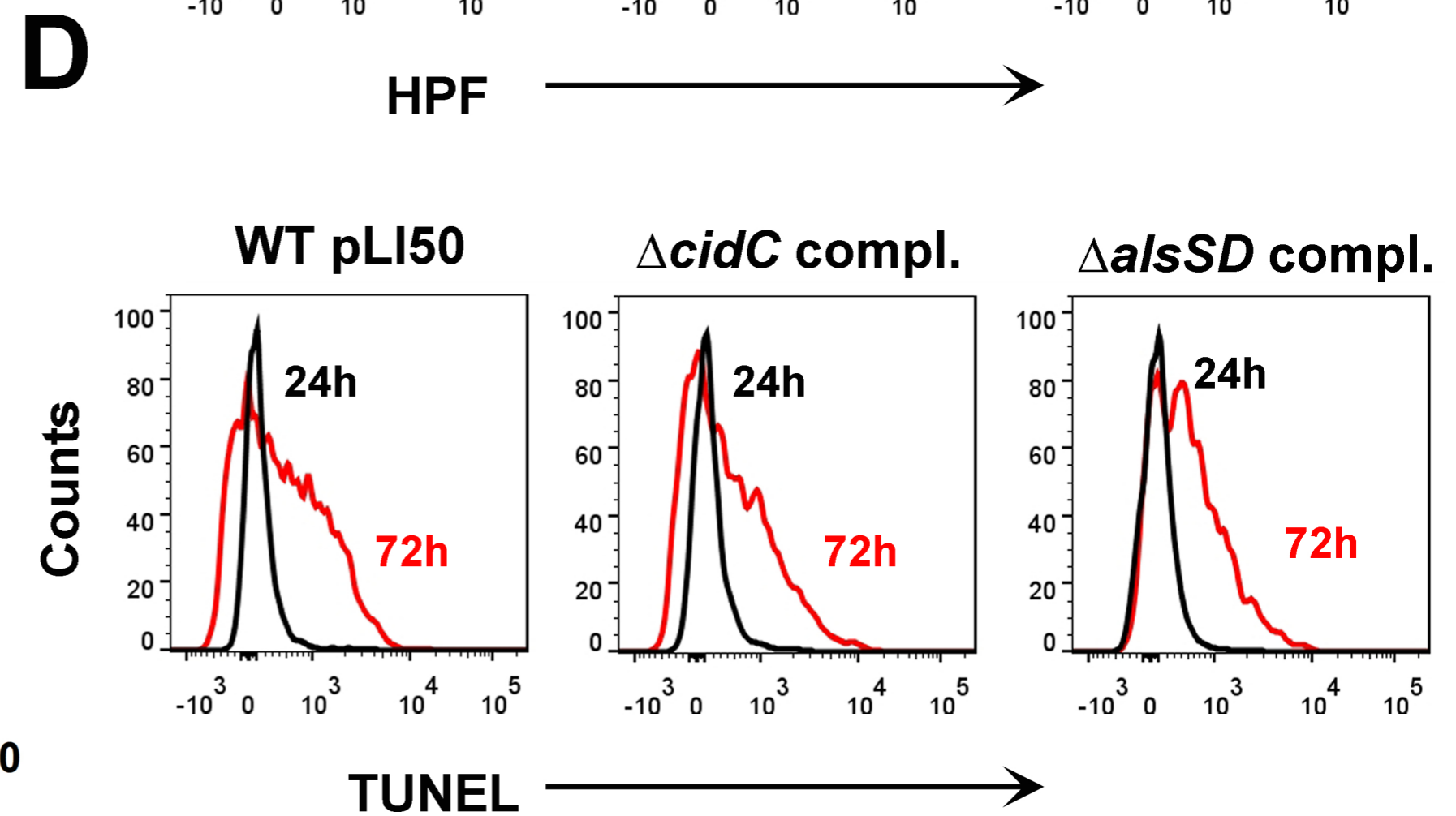

Supplement: Figure S3 — Complementation of Δ cidC and Δ alsSD mutant phenotypes. (A) Cell viabilities (cfu/ml, mean ± SD) of S. aureus UAMS-1 (WT pLI50), cidC compl. and ΔalsSD compl. were monitored every 24 h over a period of five days in TSB-35 mM glucose. Psuedocolor density plots of cells double stained with HPF/CTC (B), whole cell EPR analysis (C) and TUNEL staining (D) were carried out after 24 and 72 h growth. (PDF) [file ppat.1004205.s003.pdf]

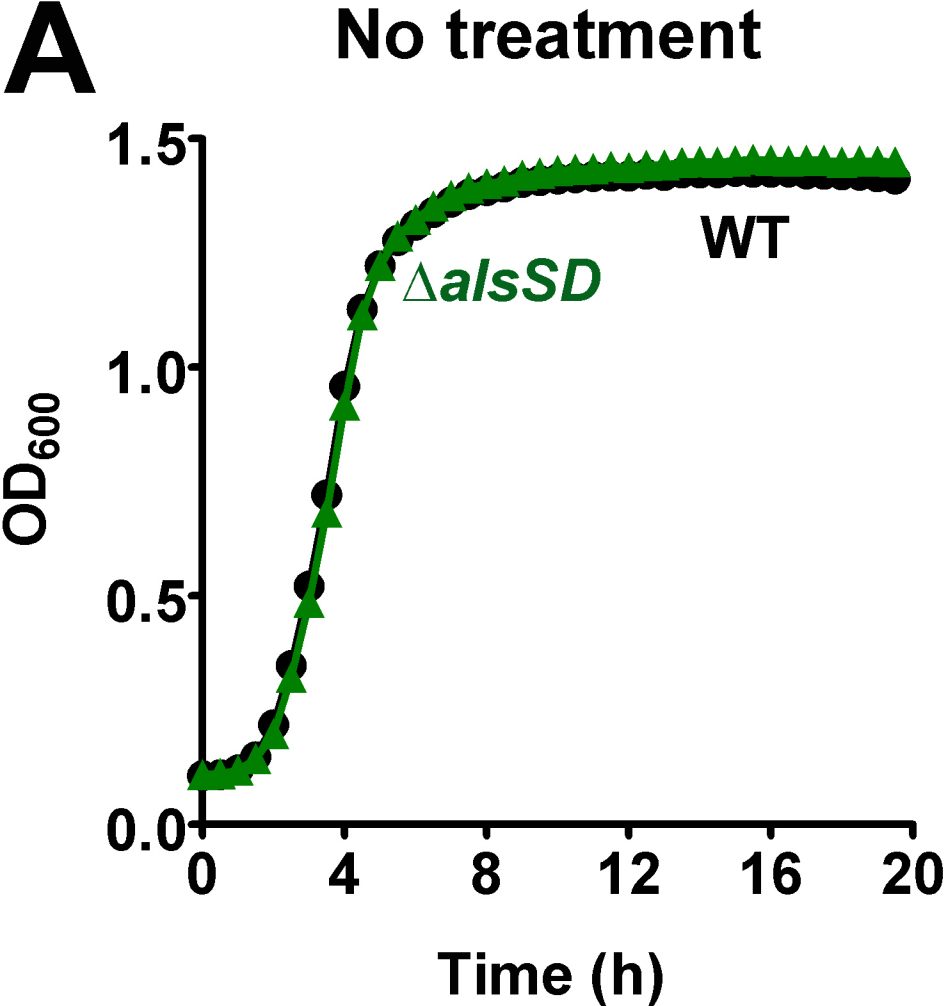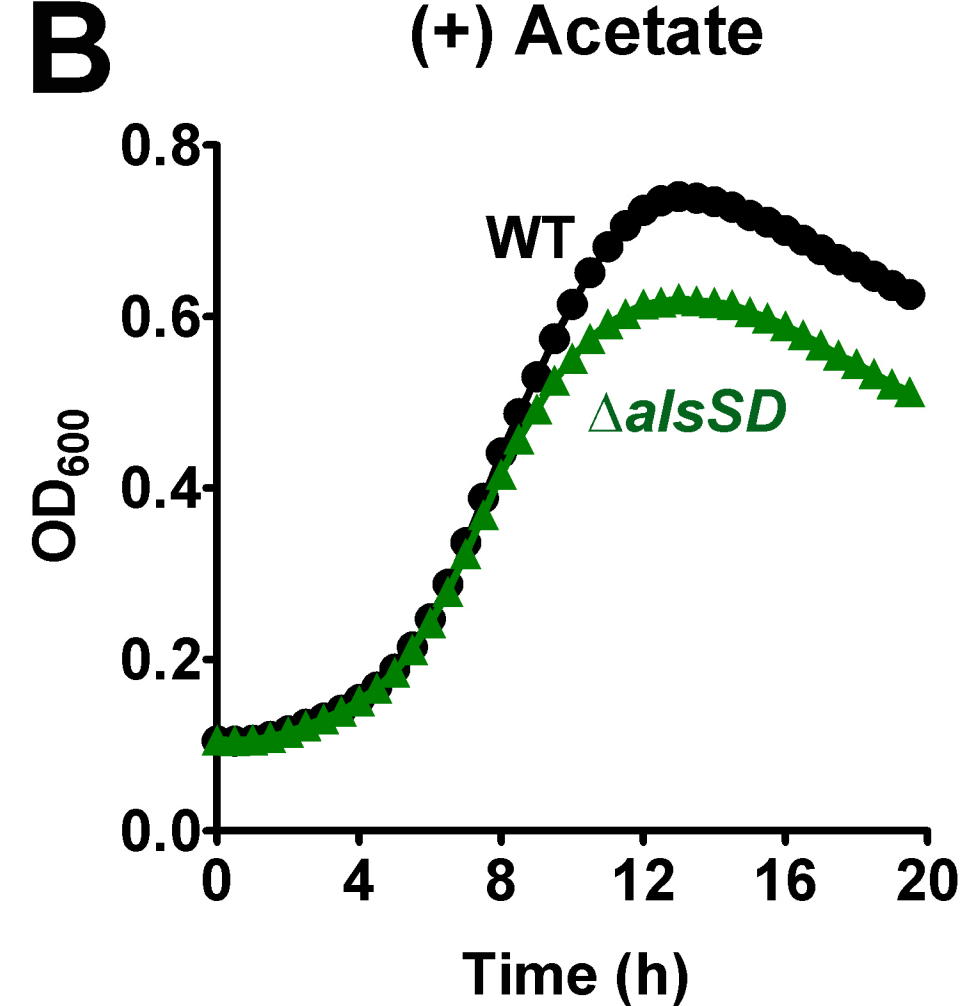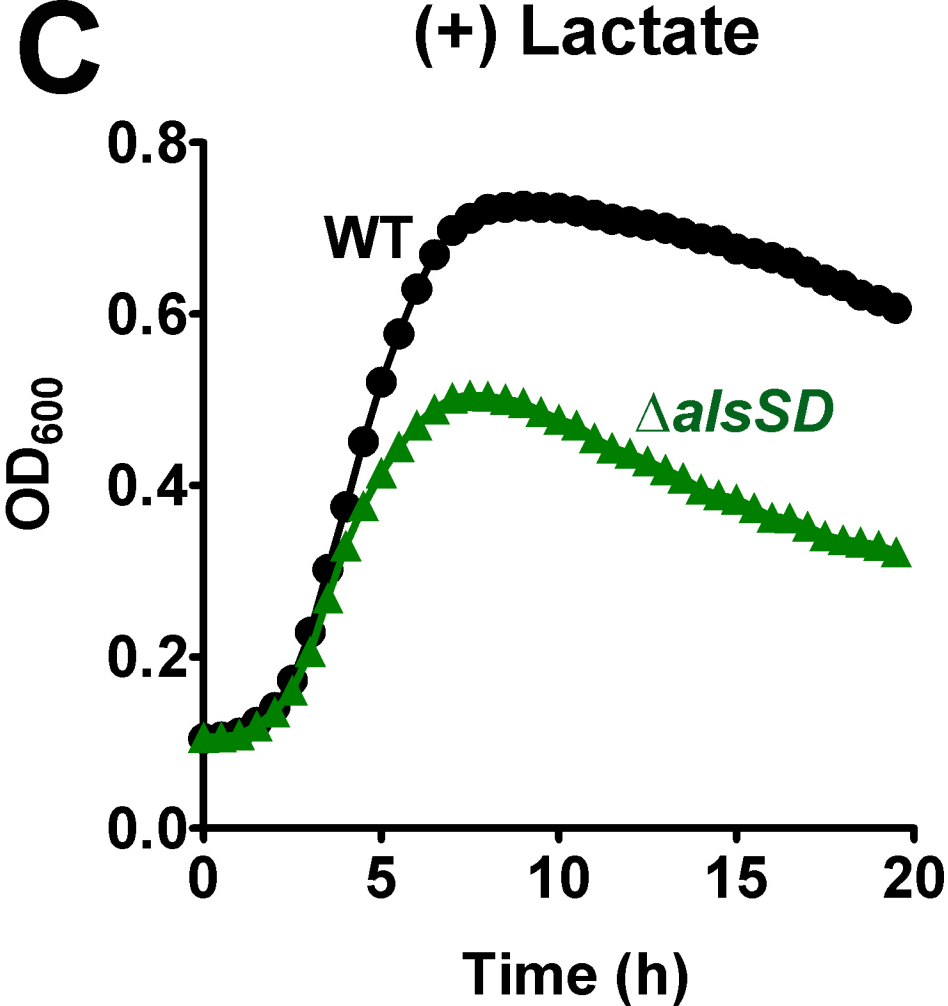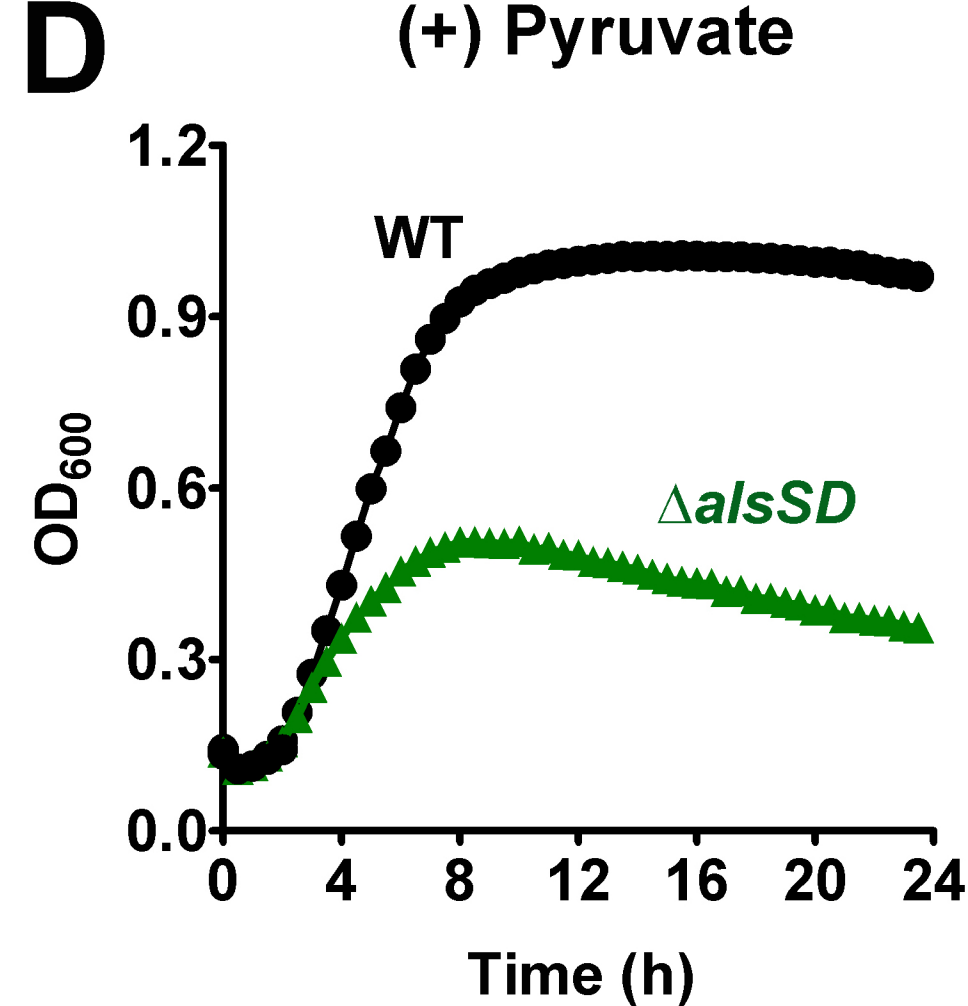

Supplement: Figure S4 — Mutation of alsSD renders S. aureus susceptible to weak acids. Overnight grown (16 to 18-hr) S. aureus cultures (WT and ΔalsSD) were resuspended to an OD600 of 0.06 in (A) TSB-35 mM glucose (untreated) or TSB-35 mM glucose supplemented with (B) acetic acid (30 mM), (C) lactic acid (40 mM) or (D) pyruvic acid (30 mM). Bacterial suspensions were dispensed into 96-well microtiter plates and grown for 24 h at 37°C in a Tecan infinite 200 spectrophotometer under maximum aeration. The absorbance signals (OD600) were recorded every 30 minutes for the entire period of growth. (PDF) [file ppat.1004205.s004.pdf]

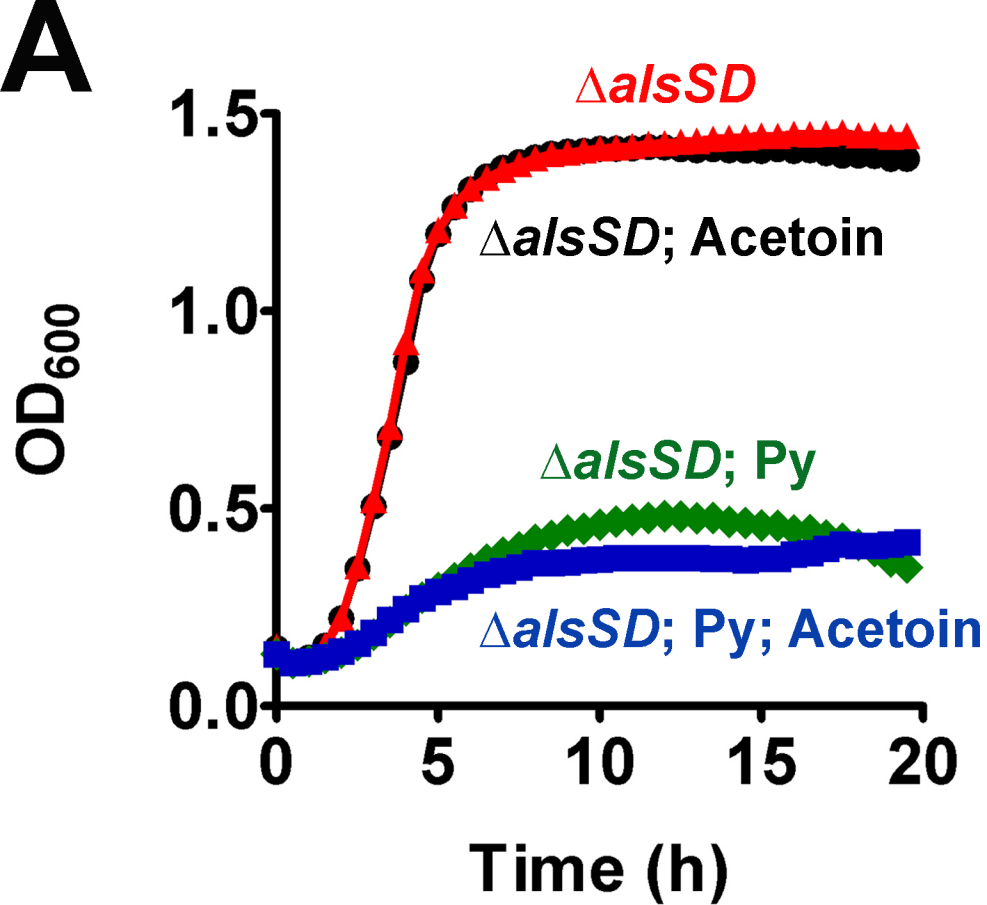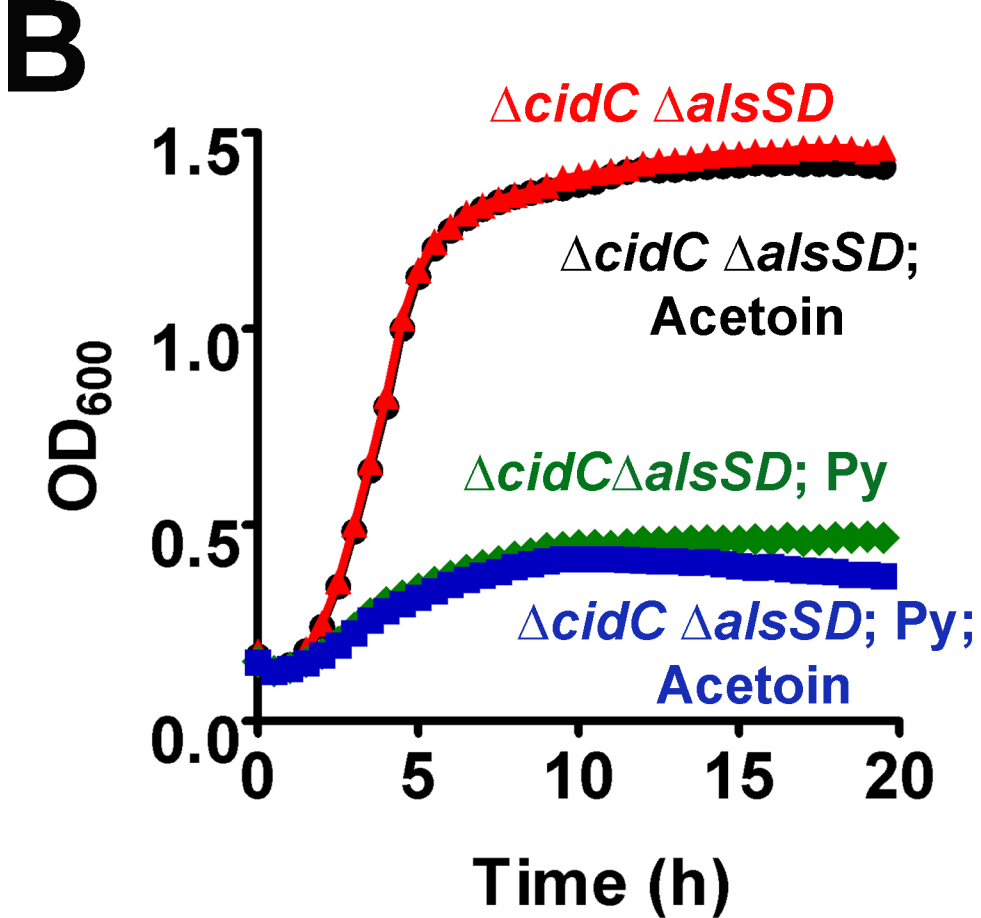

Supplement: Figure S5 — Acetoin does not rescue pyruvic acid mediated growth inhibition of alsSD mutants. Overnight grown cultures of ΔalsSD mutant (A) or ΔcidC ΔalsSD double mutant (B) were seeded to a final OD600 of 0.06 in TSB-35 mM glucose supplemented with excess acetoin (10 mM). Cultures were challenged with 30 mM pyruvic acid (Py) and growth was monitored for 24 h at 37°C in a Tecan infinite 200 spectrophotometer under maximum aeration. (PDF) [file ppat.1004205.s005.pdf]

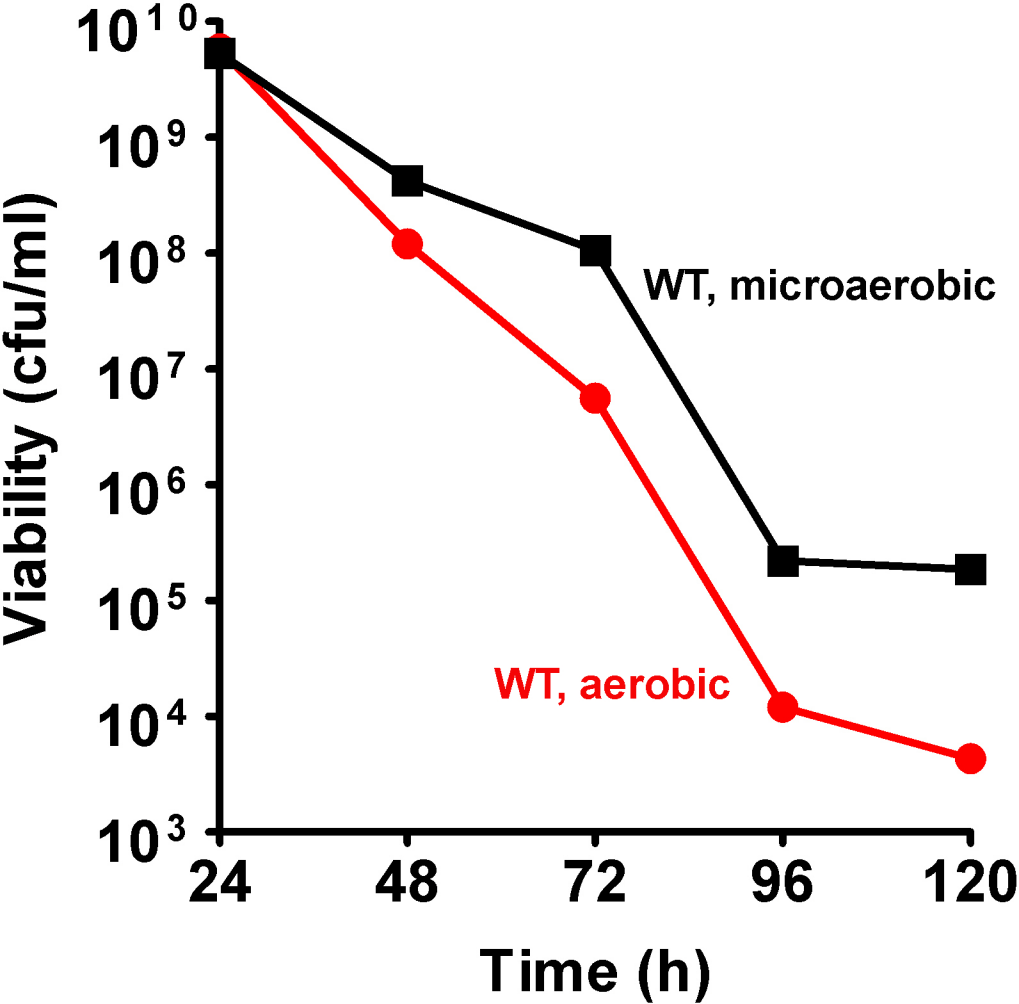

Supplement: Figure S6 — Microaerobic growth partially rescues glucose dependent stationary phase cell death. S. aureus UAMS-1 cultures aerobically grown for 24 h at 250 rpm were shifted to static conditions without agitation in a 37°C incubator for a total of 120 h. Cell viability was determined daily. (PDF) [file ppat.1004205.s006.pdf]

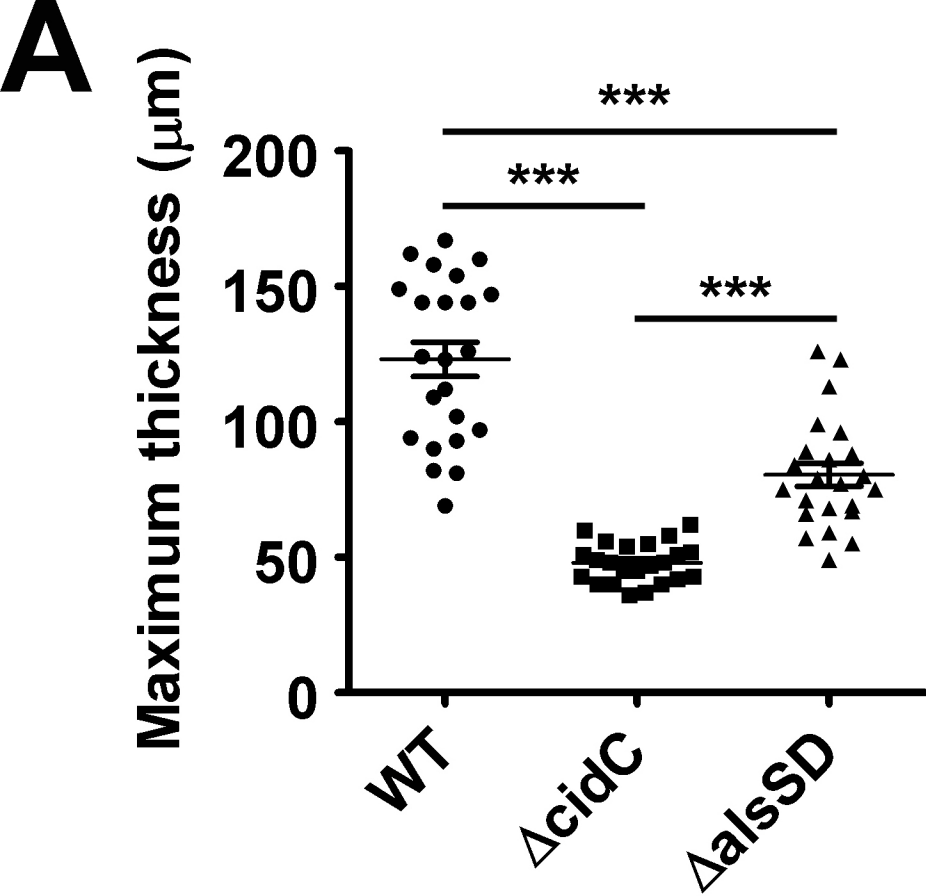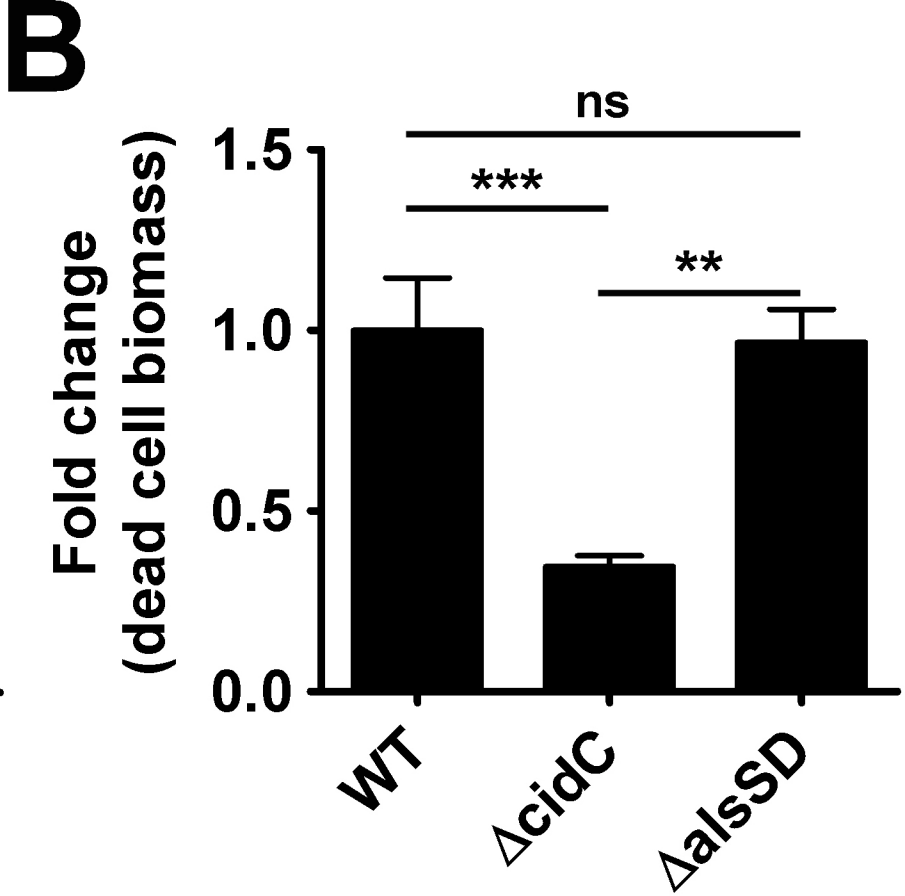

Supplement: Figure S7 — COMSTAT analysis of S. aureus UAMS-1 and metabolic mutant biofilms. (A) Maximum thickness of biofilms (B) Fold changes in dead cell biomass relative to wild-type biofilm. Fold change was determined after normalization of dead cell biomass to the total biomass (Statistical significance was assessed using one way ANOVA followed by Newman Kewl's multiple post-comparison test; ** P<0.005, *** P<0.0005). (PDF) [file ppat.1004205.s007.pdf]

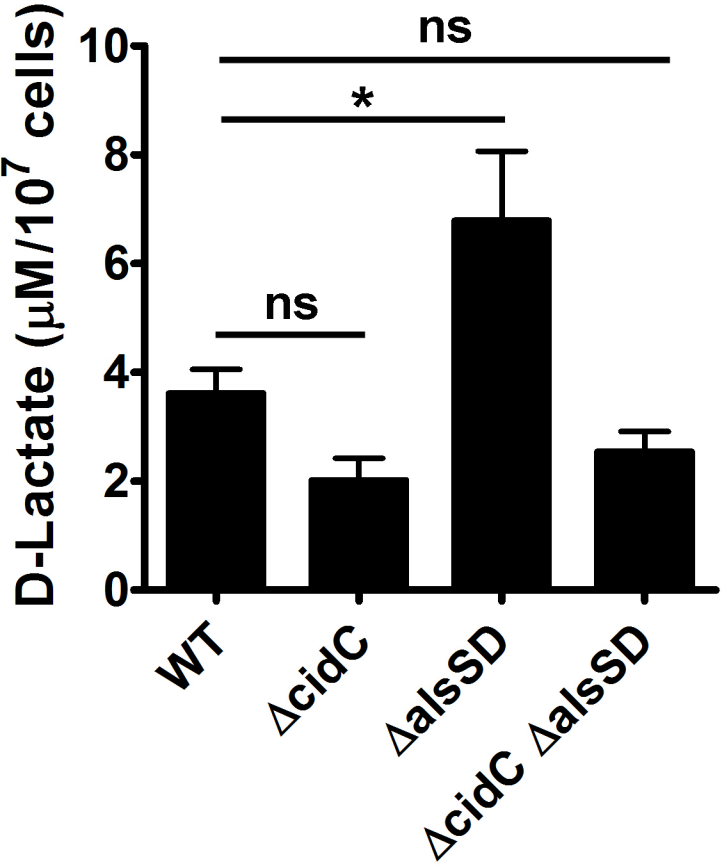

Supplement: Figure S8 — D-lactate levels in S. aureus culture supernatants. The concentration of D-lactate was determined from culture supernatants of various S. aureus strains after 24 h of growth in TSB-35 mM glucose using a commercially available kit (R-Biopharm, Germany). Statistical significance was assessed using one way ANOVA followed by Newman Kewl's multiple post-comparison test, n = 3; * P<0.05). (PDF) [file ppat.1004205.s008.pdf]

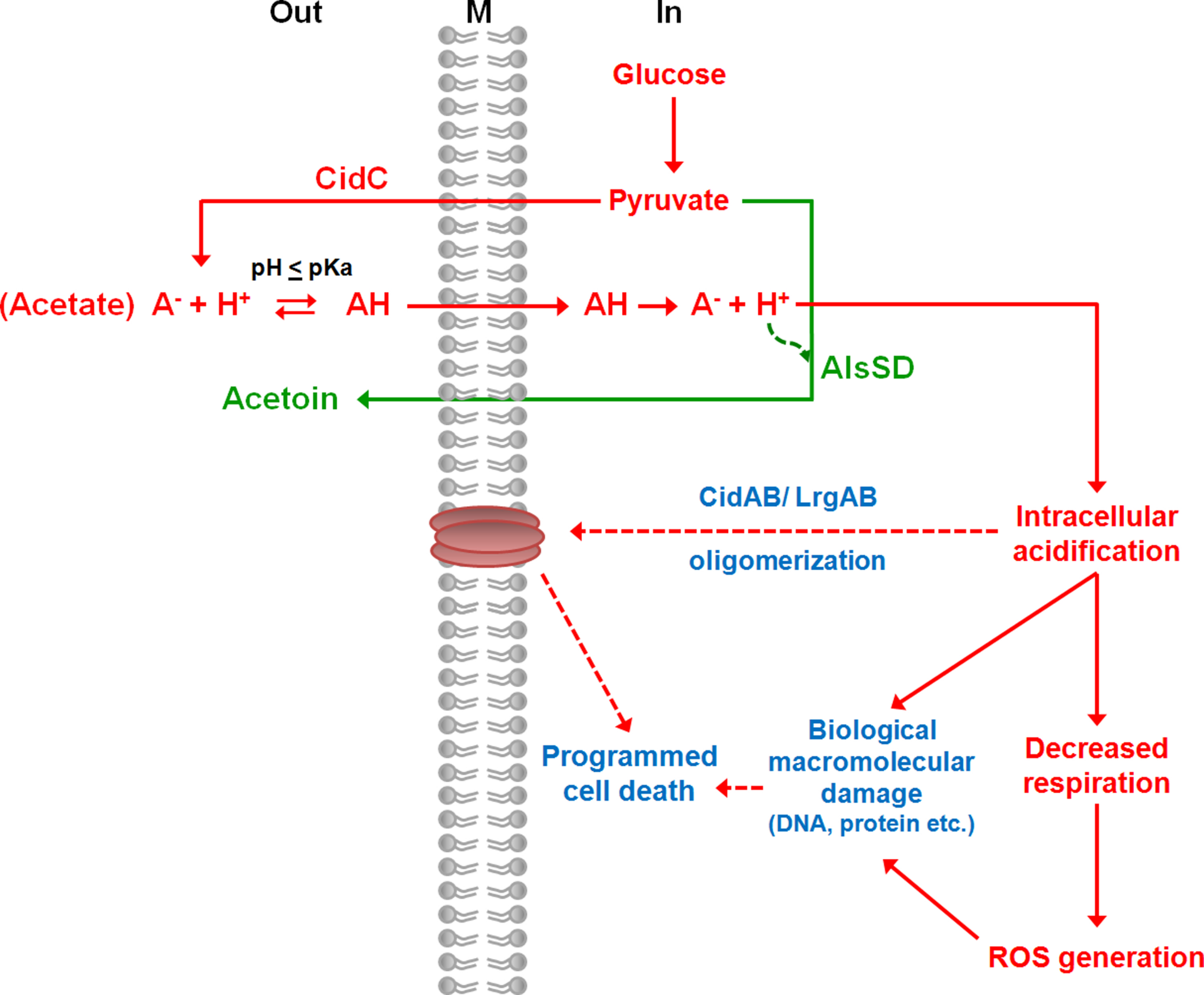

Supplement: Figure S9 — Schematic of the proposed regulation of PCD in S. aureus . Excess acetate (A−) generated by CidC activity contributes to the decrease in external pH within biofilm microcolonies. When the pHexternal approaches the pKa of acetic acid (∼4.8), the undissociated neutral form of the acid (AH) enters the cell resulting in cytoplasmic acidification. This leads to the inhibition of cellular respiration and ROS is generated in the process. Ultimately programmed cell death may result from irreparable damage to biological macromolecules like proteins, RNA and DNA exacted by acid and oxidative stress. Alternately PCD may also result from a weak acid dependent olgomerization and insertion of CidAB and/or LrgAB proteins within the membrane. To counter and limit PCD, cells limit the generation of acetate by re-routing pyruvate to acetoin (neutral) production via the AlsSD pathway. Additionally, the activity of AlsSD also results in the consumption of protons and this helps in maintenance of pH homeostasis. (PDF) [file ppat.1004205.s009.pdf]
