# Supplementary material for: A Central Role for Carbon-Overflow Pathways in the Modulation of Bacterial Cell Death
Source: PLoS Pathog. 2014 Jun 19;10(6):e1004205. doi: 10.1371/journal.ppat.1004205 (PMC4063974; doi:10.1371/journal.ppat.1004205)
Supplement: Table S1 — Flow cytometry- quadrant statistics (%). (DOC) [file ppat.1004205.s010.doc]

**Table S1 Flow cytometry-** quadrant statistics (%)

|  | **Time** | **Sample** | | **CTC^+^**  **(Quad 1)** | | **CTC^+^ HPF^+^**  **(Quad 2)** | | **HPF^+^**  **(Quad 3)** | **Unstained**  **(Quad 4)** | |
| --- | --- | --- | --- | --- | --- | --- | --- | --- | --- | --- |
|  | **24 h** | 14mM glucose WT | | **28.8 ± 1.6** | | **4.8 ± 1.2** | | **13.5 ± 3.5** | **52.9 ± 3.3** | |
|  |  | 35mM glucose MOPS WT | | **50.0 ± 1.2** | | **1.1 ± 0.3** | | **1.4 ± 0.2** | **47.6 ± 0.7** | |
|  |  | 35mM glucose WT | | **57.3 ± 0.7** | | **3.8 ± 0.7** | | **2.7 ± 0.6** | **36.1 ± 1.2** | |
|  |  | 35mM glucose *∆cidC* | | **71.6 ± 4.2** | | **0.9 ± 0.1** | | **1.1 ± 0.0** | **26.4 ± 4.3** | |
|  |  | 35mM glucose *∆alsSD* | | **62.2 ± 4.2** | | **2.3 ± 1.4** | | **1.4 ± 0.5** | **34.2 ± 5.7** | |
|  |  | 35mM glucose *∆cidC ∆alsSD* | | **60.7 ± 0.7** | | **2.7 ± 0.9** | | **2.0 ± 0.5** | **34.5 ± 1.1** | |
|  |  | WT pLI50 | | **65.4 ± 0.5** | | **6.9 ± 0.3** | | **2.8 ± 0.1** | **24.8 ± 0.3** | |
|  |  | *∆cidC* compl. | | **67.2 ± 0.7** | | **7.7 ± 1.0** | | **2.3 ± 0.3** | **22.6 ± 1.1** | |
|  |  | *∆alsSD* compl. | | **66.8 ± 0.2** | | **4.4 ± 0.5** | | **1.5 ± 0.03** | **27.1 ± 0.7** | |
|  | **72 h** | 14mM glucose WT | | **76.6 ± 0.4** | | **2.5 ± 0.5** | | **5.3 ± 1.1** | **15.6 ± 1.5** | |
|  |  | 35mM glucose MOPS WT | | **44.9 ± 1.5** | | **6.9 ± 1.8** | | **4.1 ± 1.3** | **44.2 ± 1.0** | |
|  |  | 35mM glucose WT | | **1.9 ± 0.5** | | **9.1 ± 1.6** | | **77.8 ± 3.2** | **11.2 ± 1.2** | |
|  |  | 35mM glucose *∆cidC* | | **29.2 ± 0.5** | | **5.5 ± 1.6** | | **5.6 ± 2.7** | **59.7 ± 4.5** | |
|  |  | 35mM glucose *∆alsSD* | | **2.0 ± 0.6** | | **11.5 ± 2.7** | | **77.4 ± 4.4** | **9.1 ± 1.1** | |
|  |  | 35mM glucose ∆cidC ∆alsSD | | **3.0 ± 0.4** | | **14.1 ± 1.7** | | **71.7 ± 3.9** | **11.2 ± 1.8** | |
|  |  | 35mM glucose Anaerobic MOPS WT | | **61.9 ± 9.3** | | **17.4 ± 1.0** | | **1.3 ± 0.7** | **19.4 ± 7.6** | |
|  |  | 35mM glucose Anaerobic WT | | **51.0 ± 9.4** | | **0.8 ± 0.8** | | **0.1 ± 0.1** | **48.0 ± 8.4** | |
|  |  | WT pLI50 | | **3.5 ± 0.3** | | **1.4 ± 0.1** | | **39 ± 6.3** | **55.9 ± 6.1** | |
|  |  | *∆cidC* compl. | | **5.1 ± 1.2** | | **4.0 ± 1.0** | | **43.6 ± 5.1** | **47.2 ± 3.4** | |
|  |  | *∆alsSD* compl. | | **6.7 ± 1.2** | | **2.2 ± 0.1** | | **39.7 ± 2.7** | **51.2 ± 2.5** | |
|  | | |  | |  | |  | | |  |
