# Supplementary material for: A Central Role for Carbon-Overflow Pathways in the Modulation of Bacterial Cell Death
Source: PLoS Pathog. 2014 Jun 19;10(6):e1004205. doi: 10.1371/journal.ppat.1004205 (PMC4063974; doi:10.1371/journal.ppat.1004205)
Supplement: Table S2 — Strains and plasmids. (DOC) [file ppat.1004205.s011.doc]

| **Table S2** | **Strains and plasmids** | |  | |
| --- | --- | --- | --- | --- |
|  | **Alternate designation** | **Description** | **Source** | |
| **Strains**  ***Escherichia coli*** | | | | |
| Electro-Ten-Blue |  | General plasmid maintenance strain | | Stratagene |
|  |  |  | |  |
| ***Staphylococcus aureus*** | | | | |
| RN4220 |  | Restriction deficient strain routinely used as a transformation intermediate | |  |
| UAMS-1 |  | Wild-type; MSSA; Clinical osteomyelitis isolate, *rsbU*^+^ | |  |
| ∆*cidC* | KB1058 | UAMS-1∆*cidC*::*erm*/ Em^R^ | |  |
| ∆*alsSD* | UAMS1-1489 | UAMS-1∆*alsSD*/ unmarked deletion mutant | |  |
| ∆*cidC alsSD* |  | UAMS-1∆*cidC alsSD*/ Em^R^ | | This study |
| UAMS-1 pLI50 | CYL6939 | UAMS-1 containing pLI50/ Cm^R^ | |  |
| ∆*alsSD* pLI50::*alsS* |  | UAMS-1∆*alsSD* containing pVCT-2/ Em^R^ Cm^R^ | | This study |
| ∆*cidC alsSD* pLI50::*alsS* |  | UAMS-1∆*cidC alsSD* containing pVCT-2/ Em^R^ Cm^R^ | | This study |
| ∆*cidC* compl. |  | UAMS-1∆*cidC* containing pVCT3/ Em^R^ Cm^R^ | | This study |
| ∆*alsSD* compl. | UAMS1-1551 | UAMS-1∆*alsSD* containing pLI50::*alsSD* / Em^R^/Cm^R^ | |  |
| **Plasmids** |  |  | |  |
| pLI50 |  | *E. coli*- *S. aureus* shuttle vector | |  |
| pVCT-2 |  | pLI50::*alsS* (under control of its native promoter) | | This study |
| pVCT-3 |  | pLI50::*cidC* (under control of its native promoter) | | This study |
