# Supplementary material for: A Central Role for Carbon-Overflow Pathways in the Modulation of Bacterial Cell Death
Source: PLoS Pathog. 2014 Jun 19;10(6):e1004205. doi: 10.1371/journal.ppat.1004205 (PMC4063974; doi:10.1371/journal.ppat.1004205)
Supplement: Text S1 — Supporting references. (DOC) [file ppat.1004205.s012.doc]

**Text S1. Supporting references**

1. Kreiswirth BN, Lofdahl S, Betley MJ, O'Reilly M, Schlievert PM, et al. (1983) The toxic shock syndrome exotoxin structural gene is not detectably transmitted by a prophage. Nature 305: 709-712.

2. Gillaspy AF, Hickmon SG, Skinner RA, Thomas JR, Nelson CL, et al. (1995) Role of the accessory gene regulator (*agr*) in pathogenesis of staphylococcal osteomyelitis. Infect Immun 63: 3373-3380.

3. Patton TG, Rice KC, Foster MK, Bayles KW (2005) The *Staphylococcus aureus* *cidC* gene encodes a pyruvate oxidase that affects acetate metabolism and cell death in stationary phase. Mol Microbiol 56: 1664-1674.

4. Tsang LH, Cassat JE, Shaw LN, Beenken KE, Smeltzer MS (2008) Factors contributing to the biofilm-deficient phenotype of *Staphylococcus aureus* *sarA* mutants. PLoS One 3: e3361.

5. Luong TT, Lei MG, Lee CY (2009) *Staphylococcus aureus* Rbf activates biofilm formation in vitro and promotes virulence in a murine foreign body infection model. Infect Immun 77: 335-340.

6. Lee CY, Buranen SL, Ye ZH (1991) Construction of single-copy integration vectors for *Staphylococcus aureus*. Gene 103: 101-105.
